# Primary cilia sense glutamine availability and respond via asparagine synthetase

**DOI:** 10.1038/s42255-023-00754-6

**Published:** 2023-03-06

**Authors:** Maria Elena Steidl, Elisa A. Nigro, Anne Kallehauge Nielsen, Roberto Pagliarini, Laura Cassina, Matteo Lampis, Christine Podrini, Marco Chiaravalli, Valeria Mannella, Gianfranco Distefano, Ming Yang, Mariam Aslanyan, Giovanna Musco, Ronald Roepman, Christian Frezza, Alessandra Boletta

**Affiliations:** 1 Molecular Basis of Cystic Kidney Disorders Unit, Division of Genetics and Cell Biology, IRCCS, San Raffaele Scientific Institute, Milan, Italy; 2 Ph.D Program in Molecular and Cellular Biology, Vita-Salute San Raffaele University, Milan, Italy; 3 Center for Omics Sciences, IRCCS, San Raffaele Scientific Institute, Milan, Italy; 4 MRC, Cancer Unit Cambridge, Hutchison/MRC Research Centre, University of Cambridge, Cambridge, UK; 5 Department of Human Genetics and Radboud Institute for Molecular Life Sciences, Radboud University Medical Center, Nijmegen, the Netherlands; 6 Biomolecular Nuclear Magnetic Resonance Unit, Division of Genetics and Cell Biology, IRCCS, San Raffaele Scientific Institute, Milan, Italy; 7 Present Address: Department of Biosystems Science and Engineering, ETH Zurich, Basel, Switzerland; 8 Present Address: CECAD Research Center, Cologne, Germany

**Keywords:** Organelles, Mechanisms of disease, Polycystic kidney disease, Metabolomics, Metabolism

## Abstract

Depriving cells of nutrients triggers an energetic crisis, which is resolved by metabolic rewiring and organelle reorganization. Primary cilia are microtubule-based organelles at the cell surface, capable of integrating multiple metabolic and signalling cues, but their precise sensory function is not fully understood. Here we show that primary cilia respond to nutrient availability and adjust their length via glutamine-mediated anaplerosis facilitated by asparagine synthetase (ASNS). Nutrient deprivation causes cilia elongation, mediated by reduced mitochondrial function, ATP availability and AMPK activation independently of mTORC1. Of note, glutamine removal and replenishment is necessary and sufficient to induce ciliary elongation or retraction, respectively, under nutrient stress conditions both in vivo and in vitro by restoring mitochondrial anaplerosis via ASNS-dependent glutamate generation. Ift88-mutant cells lacking cilia show reduced glutamine-dependent mitochondrial anaplerosis during metabolic stress, due to reduced expression and activity of ASNS at the base of cilia. Our data indicate a role for cilia in responding to, and possibly sensing, cellular glutamine levels via ASNS during metabolic stress.

## Abstract (web summary)

Primary cilia are shown to adjust length in response to cellular nutrient availability, with a special role for glutamine-mediated anaplerosis via the enzyme ASNS, which was found to be located at the base of cilia. Consistently, cells lacking cilia show an altered response to glutamine during metabolic stress.

## Main

Over 35 different pathologies caused by functional alterations of the primary cilium have been reported affecting various organs and physiological systems, collectively called ciliopathies^1,2^. These pathologies are classified as motile ciliopathies when they are caused by disruption of motile cilia, or sensory ciliopathies when they are caused by alterations in primary, non-motile, cilia. The kidney is frequently and severely affected in the sensory ciliopathies, presenting with a broad spectrum of phenotypes, including cyst formation, inflammation and fibrosis^1,2^.

The function of primary cilia is still largely elusive. In most cell types, it appears to act as a central hub receiving extracellular signals and integrating an intracellular response that orchestrates key cellular functions, such as proliferation and autophagy^1–4^. Cilia were also shown to regulate systemic metabolic responses via adipocyte hormone receptors, providing a possible explanation for obesity as a frequent manifestation in the ciliopathies^5,6^. Metabolic reprogramming and alterations in cellular bioenergetics are central features of polycystic kidney disease (PKD)^7–12^ the most frequent renal ciliopathy, whose progression is retarded by fasting and by fast-mimicking compounds^13–17^.

**Fig. 1:**
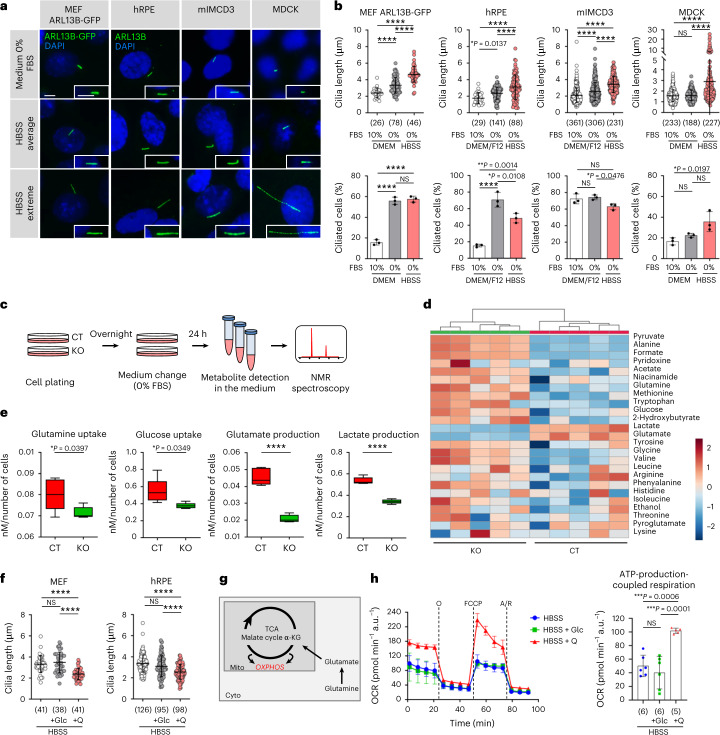
Primary cilia sense nutrient availability. **a**, Representative fluorescence images of MEFs stably expressing ARL13B-GFP, and IF images of hRPE, mIMCD3 and MDCK cells in the indicated culture conditions. Cilia (ARL13B, green), nuclei (DAPI, blue). Scale bar, 5 µm. **b**, Top: quantification of cilia length in one representative experiment in the indicated cell lines in nutrient-rich (DMEM or DMEM/F12 ± 10% FBS) or deprived (HBSS) medium. *n* indicates number of cilia whose length was measured in the same representative experiment in the indicated cell line. Bottom: percentage (%) of ciliated cells of one representative experiment in the same conditions. *n* indicates cilia percentage in three different wells per condition of the same representative experiment. **c**, Experimental design of NMR spectroscopy on MEF^Ctrl^ (CT) and MEF^*Ift88*^ (KO) conditioned medium after 24 h culture in DMEM + 0% FBS. **d**, Hierarchical clustering of extracellular metabolites assessed by NMR spectroscopy as in **c**. **e**, Box-and-whisker plots of the levels of glutamine and glucose uptake, glutamate and lactate production in MEF^Ctrl^ (CT) and MEF^*Ift88*^ (KO) cells assessed by NMR spectroscopy as in **c**, *n* = 5 biological replicates. **f**, Quantification of cilia length in one representative experiment in MEFs and hRPE in HBSS ± d-(+)-glucose (Glc) (20 mM) or l-glutamine (Q) (4 mM). *n* indicates cilia length measured in the same representative experiment. **g**, Schematic representation of glutamine utilization for OXPHOS. **h**, Left: analysis of OCR measurement of one representative experiment in MEFs after 4 h culture in HBSS ± Glc (20 mM) or Q (4 mM) in basal condition and after sequential addition of oligomycin (O), FCCP and antimycin/rotenone (A/R). Right: quantification of ATP-production-coupled respiration as in left. *n* indicates OCR measured in different wells of the same representative experiment. Box-and-whisker plots show median and minimum to maximum; data in dot and bar plots are mean ± standard deviation. Statistical analysis: Student’s unpaired two-tailed *t*-test or one-way ANOVA, followed by Tukey’s multiple comparisons test; NS, not significant, ****P < 0.0001. *n* reported in brackets. Additional replicate experiments in **b**, **f** and **h** are shown in Supplementary Fig. 1. Source data

In this Letter, on this basis we investigated whether primary cilia contribute to nutrient sensing. First, we exposed mouse embryonic fibroblasts (MEFs) to serum or nutrient deprivation for 24 h. Anti-acetylated tubulin staining showed the expected elongation of cilia in nutrient-full medium in the absence of serum and an additional remarkable elongation under nutrient deprivation (Supplementary Fig. 1a). To rule out possible artefacts of the fixation required for immunofluorescence (IF), we used live imaging on MEFs transduced with green fluorescent protein (GFP)-fused ARL13B, a ciliary-specific protein^18^ (ARL13B-GFP, Fig. 1a,b). Data confirmed that removal of nutrients strongly induces ciliary elongation, without affecting the number of ciliated cells, ruling out increased exit from the cell cycle as a trivial explanation for our findings (Fig. 1a,b). Importantly, cilia elongation upon nutrient deprivation could also be appreciated in human retinal pigment epithelial cells (hRPE), murine inner medullary collecting duct cells (mIMCD3) and Madin–Darby Canine Kidney cells (MDCK type II), all epithelial cell lines extensively used for studies on primary cilia. In these cell lines as well, cilia elongation occurred in the absence of increased percentage of ciliated cells (Fig. 1a,b).

**Extended Data Fig. 1:**
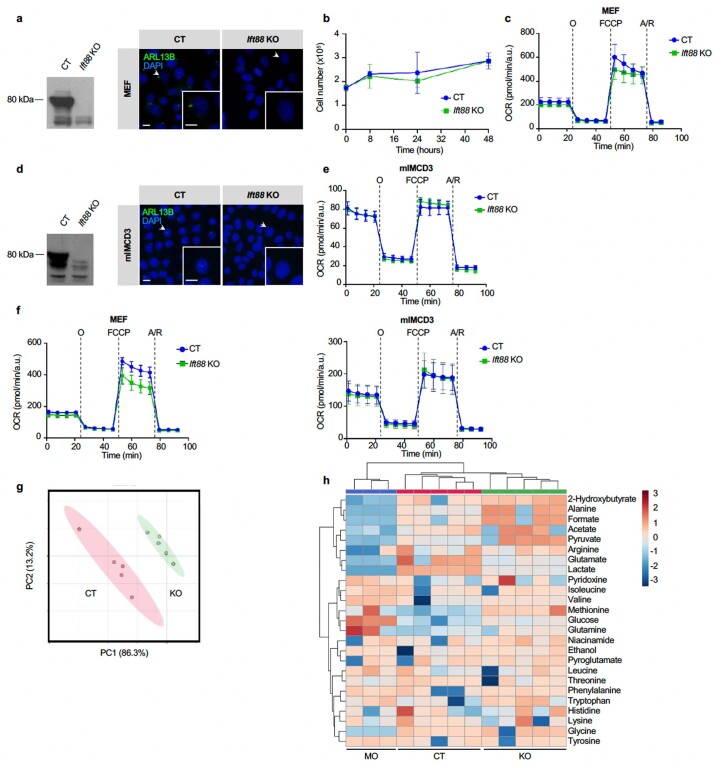
Referred to Fig. 1. **a**, Left: western blot for IFT88 of total cell lysates from MEF^*Ift88*^ and MEF^*Ctrl*^ generated by CRISPR/Cas9 technology. Right: Representative IF images of MEF^*Ift88*^ and MEF^*Ctrl*^. Cilia (ARL13B, green), nuclei (DAPI, blue). Scale bar: 10 µm. Arrows indicate cells in the insets. **b**, Growth curves in MEF^*Ift88*^ and MEF^*Ctrl*^ cultured in DMEM + 10% FBS for 8, 24, 48 hours. **c**, Analysis of OCR measurement in one representative experiment in MEF^*Ift88*^ and MEF^*Ctrl*^ cultured in DMEM + 10% FBS in basal condition and after sequential addition of oligomycin (O), FCCP, and antimycin A/rotenone (A/R). **d**, Left: western blot for IFT88 of total cell lysates from mIMCD3^*Ift88*^ and mIMCD3^*Ctrl*^ generated by CRISPR/Cas9 technology. Right: Representative IF images of mIMCD3^*Ift88*^ and mIMCD3^*Ctrl*^. Cilia (ARL13B, green), nuclei (DAPI, blue). Scale bar: 10 µm. Arrows indicate cells in the insets. **e**, Analysis of OCR measurement in one representative experiment in mIMCD3^*Ift88*^ and mIMCD3^*Ctrl*^ cultured in DMEM/F12 + 10% FBS in basal condition and after sequential addition of O, FCCP, and A/R. **f**, Left: analysis of OCR measurement in one representative experiment in MEF^*Ift88*^ and MEF^*Ctrl*^ cultured in DMEM + 0% FBS in basal condition and after sequential addition of O, FCCP, and A/R. Right: analysis of OCR measurement in one representative experiment in mIMCD3^*Ift88*^ and mIMCD3^*Ctrl*^ cultured in DMEM/F12 + 0% FBS in basal condition and after sequential addition of O, FCCP, and A/R. **g**, Principal Component analysis (PCA) of extracellular metabolites assessed by NMR spectroscopy as in Fig. 1c. **h**, Hierarchical clustering of extracellular metabolites assessed by NMR spectroscopy as in Fig. 1c, including metabolite concentrations in unconditioned medium (MO) and media conditioned by MEF^*Ift88*^ (KO) and MEF^*Ctrl*^ (CT) cells. Data in dot and bar plots are mean ± SD. Source data

We next asked whether primary cilia might be involved in the regulation of cellular metabolism. We generated MEFs and mIMCD3 cells ablated of the primary cilium by clustered regularly interspaced short palindromic repeats (CRISPR)/Cas9 inactivation of the gene encoding for the intraflagellar transport protein IFT88 (MEF^*Ift88*^ and mIMCD^*Ift88*^ hereafter), and matching controls (MEF^Ctrl^ and mIMCD^Ctrl^) leading to complete loss of cilia^14^ (Extended Data Fig. 1a–f). No overt alterations in oxygen consumption, glycolysis or proliferation rates were detected in these cells lines when grown under nutrient-rich conditions irrespectively of serum supplementation (Extended Data Fig. 1b–f). However, untargeted metabolomic by nuclear magnetic resonance (NMR) profiling of the conditioned extracellular medium in serum starvation (Fig. 1c,d and Extended Data Fig. 1g,h), revealed significant alterations in the consumption/production of metabolites in MEF^*Ift88*^ cells as compared with MEF^Ctrl^ resulting in a clear separation by principal component analysis and hierarchical clustering (Fig. 1c,d, Extended Data Tables 1 and 2, and Extended Data Fig. 1g,h). Indeed, MEF^*Ift88*^ cells displayed multiple alterations in metabolites consumption and release, including reduced glucose and glutamine consumption (Fig. 1d,e), and a significant reduction in glutamate and lactate production as compared with controls MEF^Ctrl^ (Fig. 1d,e and Extended Data Tables 1 and 2).

**Extended Data Fig. 2:**
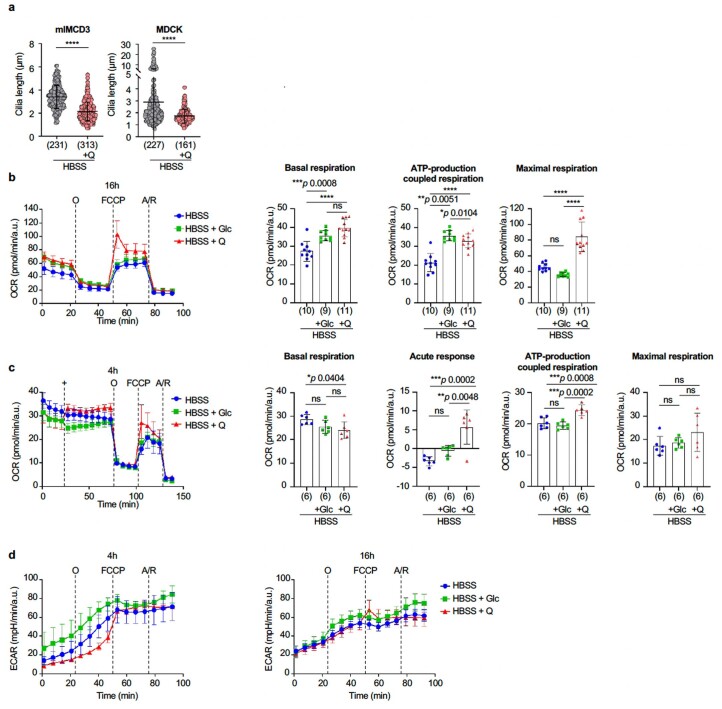
Referred to Fig. 1. **a**, Quantification of cilia length in one representative experiment in mIMCD3 and MDCK after 24 hours culture in HBSS ± L-Glutamine (Q) (4 mM). Shared control (HBSS) with Fig. 1b. **b**, Left: analysis of OCR measurement in one representative experiment in MEF after 16 hours culture in either HBSS (blue) or HBSS + D-(+)-Glucose (Glc) (20 mM) (green) or Q (4 mM) (red) in basal condition and after sequential addition of O, FCCP, and A/R. Right: quantification of basal respiration, ATP-production coupled respiration, and maximal respiration as in Left. **c**, Left: analysis of OCR measurement in one representative experiment in MEF after 4 hours culture in HBSS (blue) followed by acute injection (+) of either Glc (green) or Q (red) and after sequential addition of O, FCCP and A/R. Right: quantification of basal respiration, acute response, ATP-production coupled respiration, and maximal respiration as in Left. **d**, Left: analysis of ECAR measurement referred to Fig. 1h. Right: analysis of ECAR measurement referred to **b**. Data in dot and bar plots are mean ± SD. Statistical analysis: Student’s unpaired two-tailed *t*-test or one-way ANOVA, followed by Tukey’s multiple comparisons test; ns: not significant, ****p < 0.0001. n reported in brackets. Source data

Thus, ablation of cilia results in subtle but significant differences in the utilization of nutrients, in particular glucose and glutamine, the two main carbon sources in cells. To investigate whether the different utilization of glucose and glutamine might indicate a different sensing of these two carbon sources by cilia, we measured ciliary length upon nutrient deprivation or supplementation with glucose (25 mM) or glutamine (4 mM) (Fig. 1f). We found that glutamine, but not glucose, reversed the ciliary elongation induced by nutrient deprivation in all cell lines analysed (MEFs, hRPE, mIMCD3 and MDCK type II) (Fig. 1f and Extended Data Fig. 2a). This was interesting because, while glucose is the preferred source of energy in steady-state conditions, glutamine becomes instead the preferred source of carbon for oxidative phosphorylation (OXPHOS) and ATP production under metabolic stress conditions (such as reduced nutrient availability) (Fig. 1g) in cells^19–22^. Indeed, and in line with this, supplementation with glutamine, but not with glucose, was sufficient to drive OXPHOS and ATP production in cells exposed to nutrient deprivation (Fig. 1h and Extended Data Fig. 2b–d).

**Extended Data Fig. 3:**
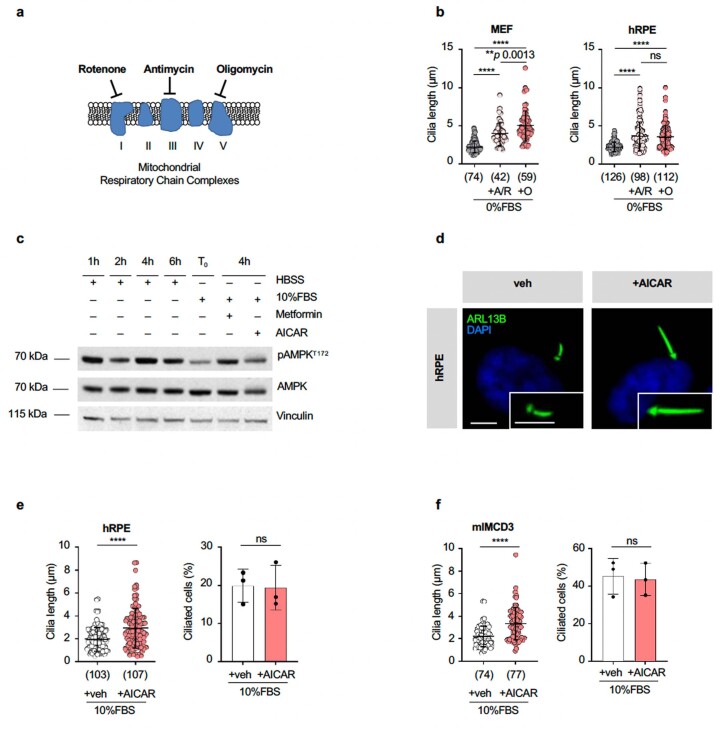
Referred to Fig. 1. **a**, Scheme of Mitochondrial Respiratory Chain Complexes targets of rotenone, antimycin, and oligomycin. **b**, Quantification of cilia length in one representative experiment in MEF and hRPE after 24 hours culture in DMEM or DMEM/F12 + 0% FBS ± either A/R or O. **c**, Western blot for pAMPK^T172^ of total cell lysates from MEF cultured for 1, 2, 4, 6 hours in HBSS or for 4 hours in either DMEM + 10% FBS ± AICAR (1 mM) or Metformin (2 mM). Vinculin is used as loading control. **d**, Representative IF images of one representative experiment after 24 hours culture in either DMEM/F12 + 10% FBS ± vehicle (veh) or AICAR. Cilia (ARL13B, green), nuclei (DAPI, blue). Scale bar: 5 µm. **e**, Left: Quantification of cilia length in one representative experiment in hRPE after 24 hours culture in the indicated conditions. Right: % of ciliated cells in one representative experiment in hRPE after 24 hours culture in the indicated conditions. **f**, Left: Quantification of cilia length in one representative experiment in mIMCD3 after 24 hours culture in the indicated conditions. Right: % of ciliated cells in one representative experiment in mIMCD3 after 24 hours culture in the indicated conditions. Data in dot and bar plots are mean ± SD. Statistical analysis: Student’s unpaired two-tailed *t*-test or one-way ANOVA, followed by Tukey’s multiple comparisons test; ns: not significant, ****p < 0.0001. n reported in brackets. Source data

Thus, we asked whether mitochondrial activity fuelled by glutamine plays a role in cilia elongation. We reasoned that, if this is the mechanism of action, inhibition of OXPHOS and mitochondrial activity should be sufficient per se to drive ciliary elongation, a finding that was previously reported in neurons^23^. Indeed, exposing cells to a mitochondrial ATP synthase inhibitor (oligomycin) or to inhibitors of complex I or complex III of the mitochondrial electron chain (rotenone and antimycin A, respectively) (Extended Data Fig. 3a) in nutrient-rich, serum-deprived medium was sufficient to increase cilia length in both MEFs and hRPE cells (Extended Data Fig. 3b). ATP availability is sensed by the energy sensor AMP-activated protein kinase (AMPK), which was strongly activated upon nutrient deprivation^24^ as expected (Extended Data Fig. 3c). Notably, pharmacological activation of AMPK using 5-aminoimidazole-4-carboxamide riboside (AICAR), a direct and specific allosteric activator of the kinase^24,25^, in cells cultured under nutrient-rich conditions (Extended Data Fig. 3c) was again sufficient to drive AMPK activity and a 50% increase in ciliary length both in hRPE and mIMCD3 cells, without affecting the percentage of ciliated cells (Extended Data Fig. 3d–f). These data taken together indicate that a reduced ATP production due to reduced OXPHOS and the consequent AMPK activation facilitates cilia elongation and that replenishment of glutamine restores ciliary length by reversing this process.

**Fig. 2:**
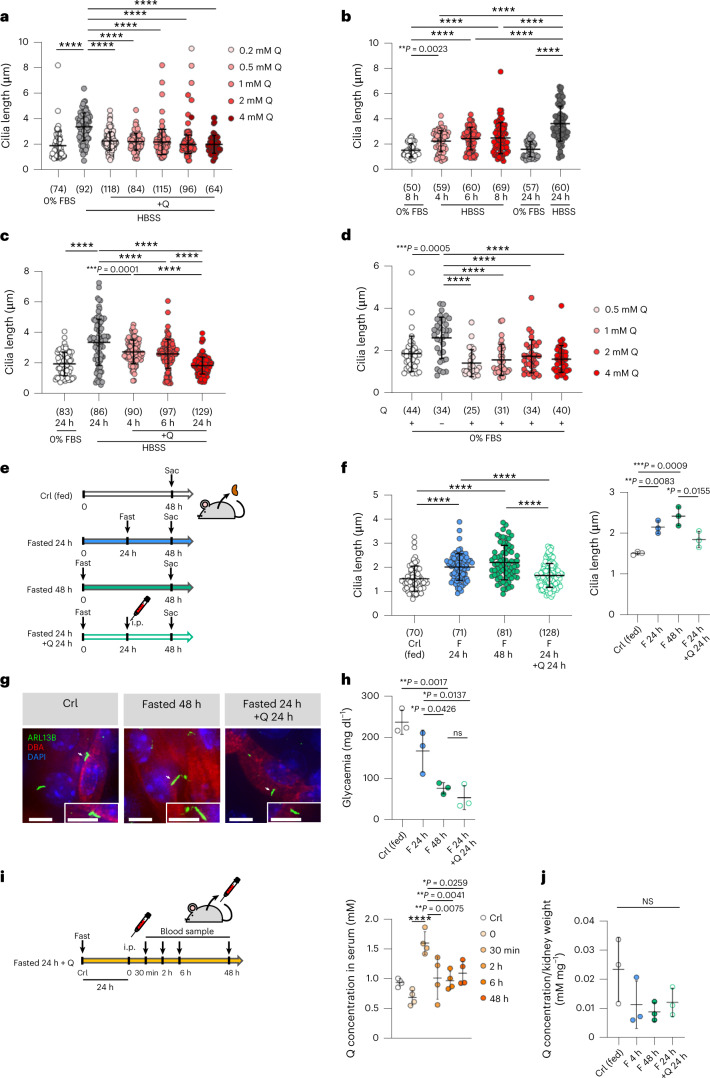
Primary cilia respond to glutamine in vitro and in vivo. **a**, Quantification of cilia length in one representative experiment in mIMCD3 after 24 h culture in 0% FBS and HBSS ± Q (0.2, 0.5, 1, 2 and 4 mM). **b**, Quantification of cilia length in one representative experiment in mIMCD3 cultured for either 8 or 24 h in 0% FBS and for 4, 6, 8 and 24 h in HBSS. **c**, Quantification of cilia length in one representative experiment in mIMCD3 cultured for 24 h in either DMEM/F12 + 0% FBS or HBSS and replenished with Q for 4, 6 and 24 h. **d**, Quantification of cilia length in one representative experiment in mIMCD3 after 24 h culture in DMEM/F12 + 0% FBS ± Q (0.5, 1, 2 and 4 mM). **e**, Experimental design of mice treatment: Crl (fed), fasted (F) for 48 or 24 h, fasted for 24 h + 24 h treatment with 800 mg kg^−1^ of Q. Sac = Sacrifice. **f**, Left: distribution of the individual cilia length in kidney sections of one representative experiment of mice treated as in **e**. Right: average ciliary length calculated from three independent experiments as in **e**. **g**, Representative IF images out of three independent experiments of kidney sections of mice Crl (fed), fasted 48 h, and fasted 24 h + Q 24 h. Cilia (ARL13B, green), DBA^+^ tubular cells (DBA, red), nuclei (DAPI, blue). Scale bar, 5 µm. **h**, Average glycaemic values from three independent experiments in mice treated as in **e**. **i**, Left: experimental design of mice treatment: Crl (fed), fasted for 24 h, and fasted for 24 h + treatment with 800 mg kg^−1^ of Q. Right: average of Q concentration in the serum of mice treated as in left from four independent experiments. **j**, Average of Q concentration normalized on the kidney weight in mice treated as in **e** from three independent experiments. Data in dot and bar plots are mean ± standard deviation. Statistical analysis: one-way ANOVA, followed by Tukey’s multiple comparisons test; NS, not significant, ****P < 0.0001. *n* reported in brackets. Additional replicate experiments in **a**–**d** are shown in Supplementary Fig. 3. Source data

Notably cilia elongation upon nutrient stress was reversed by glutamine in a dose-dependent manner and occurred at a low concentration of 0.2 mM (Fig. 2a). Furthermore, the ciliary response to nutrients is rapid, as cilia elongation could be appreciated at 8 h (Fig. 2b), while glutamine replenishment shortened the cilium already at 4 h (Fig. 2c). Finally, removal of glutamine from an otherwise full medium was sufficient to drive cilia elongation (Fig. 2d). Thus, glutamine regulates ciliary length in vitro.

**Extended Data Fig. 4:**
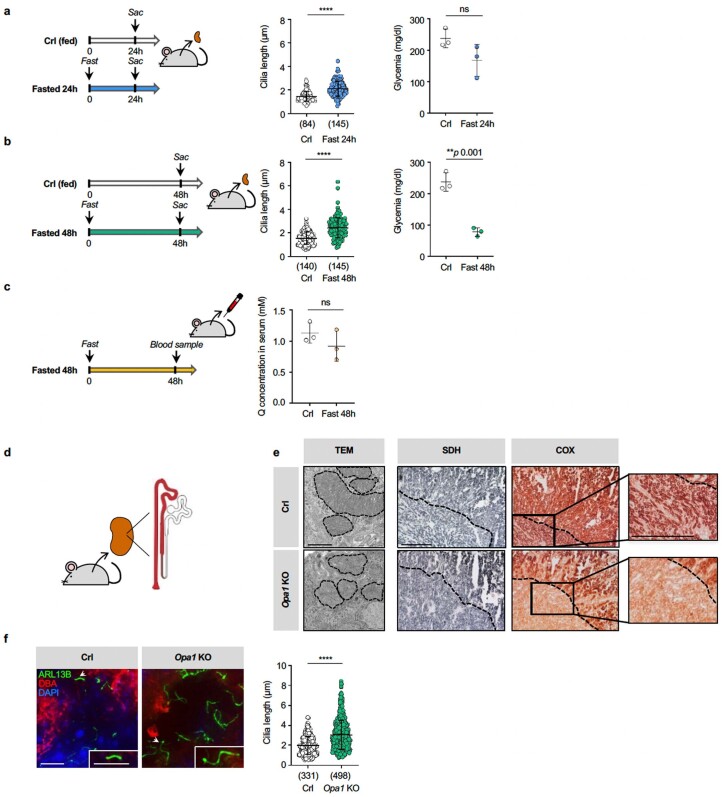
Referred to Fig. 2. **a**, Left: experimental design of mice treatment: Crl (Fed), Fasted for 24 hours. Center: quantification of cilia length in one representative experiment in kidney sections of mice treated as in Left. Right: average from 3 independent experiments of glycaemia values in mice treated as in Left. **b**, Left: experimental design of mice treatment: Crl (Fed), Fasted for 48 hours. Center: quantification of cilia length in one representative experiment in kidney sections of mice treated as in Left. Right: average from 3 independent experiments of glycaemia values in mice treated as in Left. **c**, Left: experimental design of mice treatment: Crl (Fed), Fasted for 48 hours. Right: average from 3 independent experiments of Q concentration in the serum of mice treated as in Left. **d**, Scheme of renal tubular segment with Cre-recombinase driven inactivation of *Opa1* gene. **e**, Left: Transmission Electron Microscopy (TEM) images of kidney sections of control (Crl) and *Opa1* KO mice at P2. Scale bar: 1 µm. Dashed Dots indicate mitochondria. Right: Enzymatic activity staining for Succinate Dehydrogenase (SDH) and Cytochrome c Oxidase (COX) of kidney sections of Crl and *Opa1* KO mice at P30 with zoom in. Dashed dots indicate medulla edge. Scale bar: 500 µm. **f**, Left: representative IF images of kidney sections of Crl and *Opa1* KO mice at P30. Right: quantification of cilia length in one representative experiment of DBA + tubular cells in kidney sections of Crl and *Opa1* KO mice at P30. Cilia (ARL13B, green), DBA + tubular cells (DBA, red) nuclei (DAPI, blue). Scale bar: 5 µm. Arrows indicate cilia in the insets. Data in dot and bar plots are mean ± SD. Statistical analysis: Student’s unpaired two-tailed *t*-test; ns: not significant, ****p < 0.0001. n reported in brackets. Source data

We then assessed whether the process could be observed in vivo. To test this, we analysed primary cilia length in renal tubular epithelial cells (labelled with Dolichos biflorus agglutinin, DBA) in mice fasted for 24 or 48 h (Fig. 2e–h and Extended Data Fig. 4a,b). As evidence of the fasting state, mice showed reduced glycaemic values (168 mg dl^−1^ at 24 h, and 77 mg dl^−1^ at 48 h) compared with controls (237 mg dl^−1^) (Fig. 2h and Extended Data Fig. 4a,b). Fasted mice showed a significant increase in ciliary length compared with control, fed mice, both at 24 and at 48 h of fasting (Fig. 2e–h and Extended Data Fig. 4a,b). Next, we performed intraperitoneal (i.p.) injection of 800 mg kg^−1^ of glutamine for 24 or 40 h (Fig. 2e–h), and found that this reversed the fasting-induced elongation of primary cilia (Fig. 2e–h), in the absence of an increased glycaemia (Fig. 2h). Of interest, fasting caused only a minimal decrease of circulating glutamine (Extended Data Fig. 4c), in line with previous literature^26^. Pharmacokinetic analysis revealed a peak of concentration 30 min after injection of glutamine, which achieved 1.5 mM, just 1.5 times the baseline plasma concentration of glutamine, to then return to baseline levels by 48 h (Fig. 2i). The concentration of glutamine in total kidney lysate evaluated at sacrifice revealed quite drastically decreased levels upon fasting, minimally increased after 24 h from the injection of glutamine (Fig. 2j), probably reflecting a similar kinetic as plasma glutamine (Fig. 2i). These data indicate that primary cilia elongate in response to nutrient stress and shorten in response to glutamine levels also in vivo. Finally, given the role that mitochondrial activity plays in the regulation of ciliary length in response to glutamine, we next examined ciliary length in mice carrying kidney-specific inactivation of the gene *Opa1* (ref. ^27^) (Extended Data Fig. 4d) in the same renal tubules that responded to fasting/glutamine in the previous experiment (distal and collecting ducts, DBA positive). These mutants display reduced fusion and cristae formation in mitochondria (Cassina et al., unpublished), along with a severe impairment of OXPHOS (Extended Data Fig. 4e). We found that DBA-positive renal tubules displayed a very prominent cilia elongation in the *Opa1* mutants. Thus, in line with our data above and with a recent in vitro study on astrocytes^28^, impairment of mitochondrial activity in renal epithelia in vivo results in cilia elongation (Extended Data Fig. 4f).

**Fig. 3:**
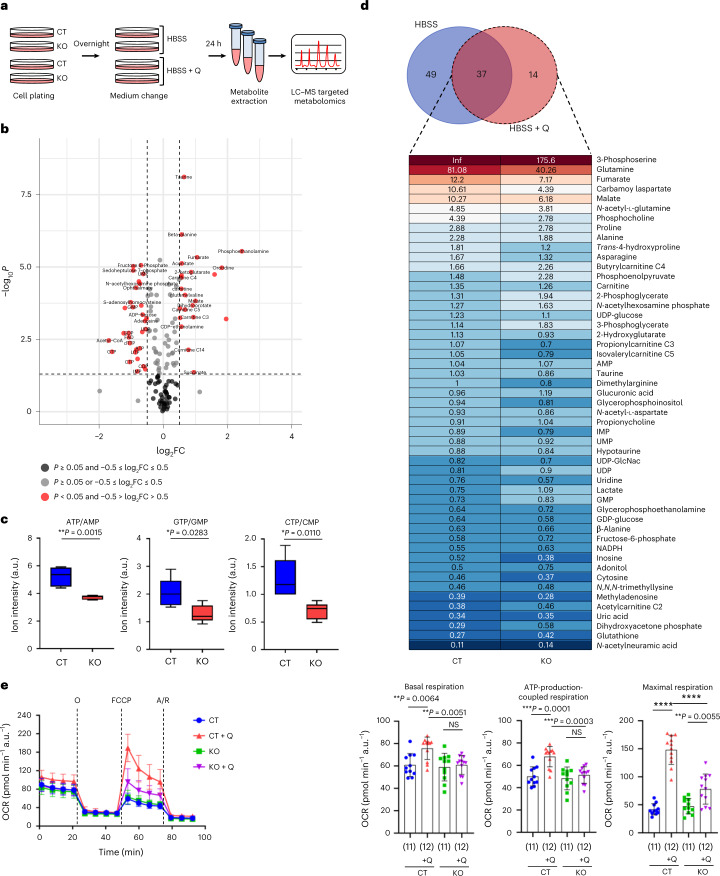
Defective response to glutamine under stress conditions in cilia-ablated cells. **a**, Experimental design of LC–MS targeted metabolomics in MEF^Ctrl^ (CT) and MEF^*Ift88*^ (KO) after 24 h culture in HBSS ± l-glutamine (Q) (4 mM). **b**, Volcano plot of metabolites in KO versus CT cultured in HBSS for 24 h as assessed by LC–MS targeted metabolomics as in **a**. FC, fold change. Black dots: *P* ≥ 0.05 and −0.5 ≤ log_2_FC ≤ 0.5; grey dots: *P* ≥ 0.05 or −0.5 ≤ log_2_FC ≤ 0.5; red dots: *P* < 0.05 and −0.5 > log_2_FC > 0.5. **c**, Box-and-whisker plots of ATP/AMP, GTP/GMP and CTP/CMP ratio in MEF^Ctrl^ and MEF^*Ift88*^ cells in HBSS for 24 h assessed by LC–MS targeted metabolomics as in **a**, *n* = 5 biological replicates. **d**, Top: Venn diagram showing the metabolites that significantly change in KO versus CT in HBSS and HBSS + Q. Light blue: 49 metabolites that change in KO versus CT only in HBSS. Rose: 14 metabolites that change in KO versus CT only in HBSS + Q. Purple: 37 metabolites that change in KO versus CT in HBSS and HBSS + Q. Bottom: Heat map showing the FC of HBSS + Q versus HBSS, in CT and KO, of the statistically different 51 metabolites. The 14 metabolites that change in KO versus CT in HBSS + Q are expressed in bold. Inf = Infinite. **e**, Left: analysis of OCR measurement of one representative experiment in MEF^Ctrl^ (CT) and MEF^*Ift88*^ (KO) after 4 h culture in either HBSS (CT, blue; KO, green) or HBSS + l-glutamine (Q) (4 mM) (CT, red; KO, purple) in basal condition and after sequential addition of oligomycin (O), FCCP and antimycin A/rotenone (A/R). Right: quantification of basal respiration, ATP-production-coupled respiration and maximal respiration as in left. Box-and-whisker plots show median and minimum to maximum; data in bar plots are mean ± standard deviation. Statistical analysis: Student’s unpaired two-tailed *t*-test or one-way ANOVA, followed by Tukey’s multiple comparisons test; NS, not significant, ****P < 0.0001. *n* reported in brackets. Additional replicate experiment in **e** is shown in Supplementary Fig. 5. Source data

**Extended Data Fig. 5:**
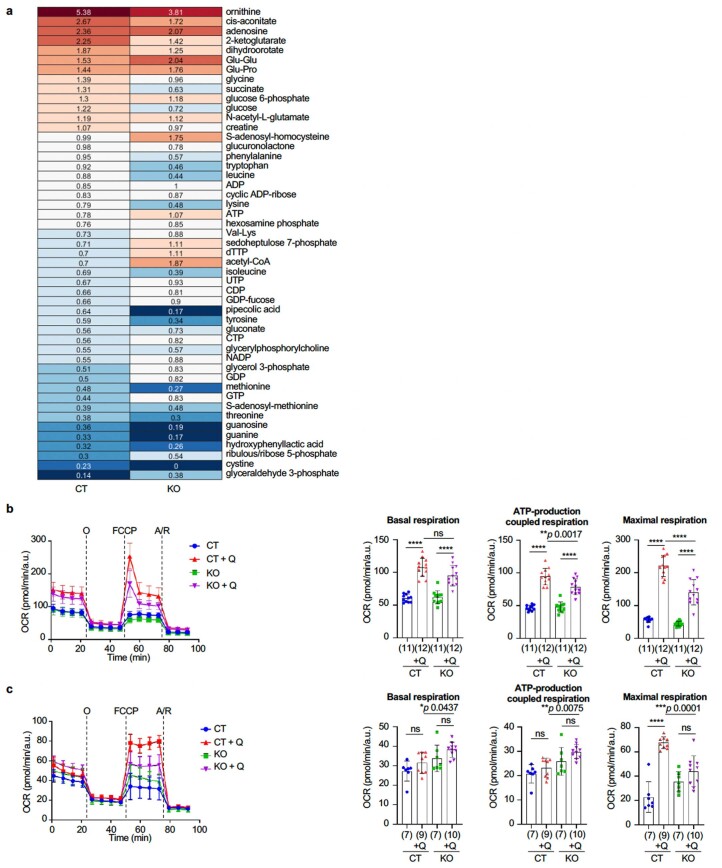
Referred to Fig. 3. **a**, Heat-map showing the fold-change of HBSS + L-Glutamine (Q) *vs* HBSS, in MEF^*Ift88*^ and MEF^*Ctrl*^, of the 49 metabolites that change in a statistically significant way in the comparison between KO *vs* CT in HBSS. **b**, Left: analysis of OCR measurement in one representative experiment in MEF^*Ift88*^ and MEF^*Ctrl*^ after 24 hours culture in either HBSS (CT: blue, KO: green) or HBSS + Q (4 mM) (CT: red, KO: purple) in basal condition and after sequential addition of oligomycin (O), FCCP, and antimycin A/rotenone (A/R). Right: quantification of basal respiration, ATP-production coupled respiration, and maximal respiration as in Left. **c**, Left: analysis of OCR measurement in one representative experiment in mIMCD3^*Ift88*^ and mIMCD3^*Ctrl*^ cells after 4 hours culture in either HBSS (CT: blue, KO: green) or HBSS + Q (4 mM) (CT: red, KO: purple), in basal condition and after sequential addition of O, FCCP, and A/R. Right: quantification of basal respiration, ATP-production coupled respiration, and maximal respiration as in Left Data bar plots are mean ± SD. Statistical analysis: one-way ANOVA, followed by Tukey’s multiple comparisons test; ns: not significant, ****p < 0.0001. n reported in brackets. Source data

We next investigated whether cilia-deficient cells present alterations in the response to metabolic stress^20–22^. To this end, we performed targeted metabolomics by liquid chromatography–mass spectrometry (LC–MS) on MEF^*Ift88*^ and MEF^Ctrl^ upon metabolic stress exposure (Hank’s Balanced Salt Solution, HBSS) or glutamine supplementation (HBSS + 4 mM Q) (Fig. 3a). Indeed, 49 of the 137 metabolites were identified as significantly changed between MEF^*Ift88*^ and MEF^Ctrl^ under HBSS exposure (Fig. 3b–d). Notably, among the most prominent changes are the ratios ATP/AMP, GTP/GMP and CTP/CMP indicating an alteration in the energy charge in response to nutrient deprivation in cilia-deficient cells (Fig. 3c). Presence of glutamine in the nutrient deprivation medium resulted in partial or complete rescue of such metabolites in the MEF^*Ift88*^ cells as compared with the MEF^Ctrl^ with the notable exception of 14 metabolites, which changed only under HBSS + Q conditions (Fig. 3d). Among these, tricarboxylic acid (TCA) cycle intermediates significantly changed in MEF^*Ift88*^ (Fig. 3d and Extended Data Fig. 5a), suggesting a possible impairment of the TCA cycle. In line with this, mitochondrial function in both MEF^*Ift88*^ and mIMCD^*Ift88*^ grown in HBSS or HBSS supplemented with 4 mM glutamine revealed that cilia-deficient cells displayed reduced oxygen consumption rate (OCR) upon glutamine replenishment in nutrient starvation (Fig. 3e and Extended Data Fig. 5b,c). Thus, cells lacking cilia utilize reduced levels of glutamine and display defective mitochondrial respiration under metabolic stress conditions.

**Extended Data Fig. 6:**
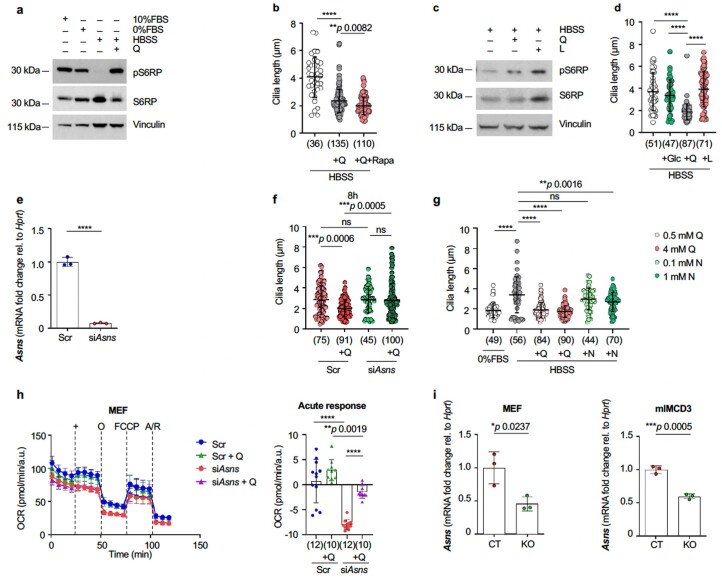
Referred to Fig. 4. **a**, Western blot for pS6RP^S235/236^ of total cell lysates from mIMCD3 cells after 24 hours culture in either DMEM/F12 ± 10% FBS or HBSS ± L-Glutamine (Q) (4 mM). Vinculin is used as loading control. **b**, Quantification of cilia length in one representative experiment in mIMCD3 cells after 24 hours culture in either HBSS ± Q or HBSS + Q + Rapamycin (100 nM). **c**, Western blot for pS6RP^S235/236^ of total cell lysates from mIMCD3 cells after 24 hours culture in either HBSS ± Q or L-Leucine (L) (0.5 mM). **d**, Quantification of cilia length in one representative experiment in mIMCD3 cells after 24 hours culture in either HBSS ± D-(+)-Glucose (Glc) (20 mM) or Q (4 mM) or L (5 mM). **e**, qRT-PCR analysis of *Asns* expression in one representative experiment in mIMCD3 transiently knocked down for *Asns* (si*Asns*) relative to control (Scr). **f**, Quantification of cilia length in one representative experiment in mIMCD3 after 8 hours culture in HBSS or HBSS + Q (4 mM). **g**, Quantification of cilia length in one representative experiment in mIMCD3 after 24 hours culture in either DMEM/F12 + 0% FBS or HBSS or HBSS + Q (0.5 or 4 mM) or HBSS + L-Asparagine (N) (0.1 or 1 mM). **h**, Left: analysis of OCR measurement in one representative experiment in si*Asns* MEF cells compared to Scr after 4 hours in HBSS (Scr: blue, si*Asns*: red) followed by acute injection (+) of Q (Scr: green, si*Asns*: purple), in basal condition and after sequential addition oligomycin (O), FCCP, and antimycin A/rotenone (A/R). Right: quantification of acute response as in Left. **i**, qRT-PCR analysis of *Asns* expression in one representative experiment in MEF^*Ift88*^ and mIMCD3^*Ift88*^ relative to MEF^*Ctrl*^ and mIMCD3^*Ctrl*^. Data in dot and bar plots are mean ± SD. Statistical analysis: Student’s unpaired two-tailed *t*-test or one-way ANOVA, followed by Tukey’s multiple comparisons test; ns: not significant, ****p < 0.0001. n reported in brackets. Source data

In search for a mechanism for our findings, we first hypothesized that the mechanistic target of rapamycin complex I (mTORC1) (ref. ^29^) might play a role, as the cascade was previously implicated in regulation of ciliary function^29,30^ and glutamine is a known activator of the pathway^29,30^. Indeed, mTORC1 was inhibited in nutrient deprivation and re-activated by glutamine replenishment as expected (Extended Data Fig. 6a). However, rapamycin did not prevent the glutamine-induced rescue of cilia elongation (Extended Data Fig. 6b). Furthermore, leucine, another amino acid known to induce mTORC1 activity^29^ (Extended Data Fig. 6c), had no effect on ciliary length (Extended Data Fig. 6d). Thus, the mechanism appears to be mTORC1 independent.

**Fig. 4:**
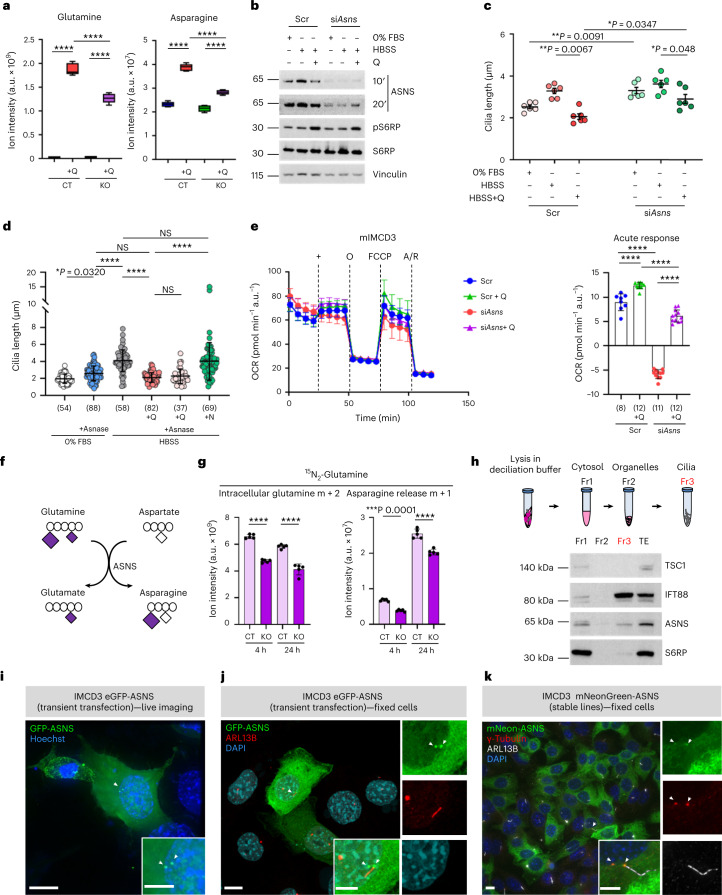
The glutamine response of cilia is mediated by the enzyme ASNS. **a**, Box-and-whisker plots of the levels of intracellular glutamine and asparagine in MEF^Ctrl^ and MEF^*Ift88*^ cells in HBSS ± l-glutamine (Q) assessed by LC–MS targeted metabolomics as in Fig. 3a, *n* = 5 biological replicates. **b**, Western blot for ASNS and pS6RP^S235/236^ of total cell lysates from mIMCD3 transiently knocked down for *Asns* (si*Asns*) compared with control (Scr) after 24 h in either 0% FBS or HBSS ± Q (4 mM). **c**, Average cilia length values from six independent experiments (in each counting >50 cilia per condition) in si*Asns* mIMCD3 compared with Scr after 24 h culture in either DMEM/F12 + 0% FBS or HBSS ± Q. **d**, Quantification of cilia length of one representative experiment in mIMCD3 after 24 h in either 0% FBS ± Asnase (5 U ml^−1^), HBSS ± Q, or Q and Asnase, or asparagine (N) (0.1 mM). **e**, Left: analysis of OCR measurement of one representative experiment in si*Asns* mIMCD3 compared with Scr after 4 h culture in HBSS (Scr, blue; si*Asns*, red) followed by acute injection (+) of Q (Scr, green; si*Asns*, purple), in basal condition and after sequential addition of oligomycin (O), FCCP and antimycin A/rotenone (A/R). Right: quantification of acute response as in left. **f**, Scheme of ^15^N_2_-glutamine usage by ASNS. **g**, Levels of intracellular glutamine m + 2 and asparagine m + 1 release assessed by ^15^N_2_-glutamine labelled LC–MS targeted metabolomics in MEF^Ctrl^ (CT) and MEF^*Ift88*^ (KO) at 4 and 24 h. *n* = 5 biological replicates. **h**, Top: scheme of cilia enrichment by subcellular fractionation. Bottom: representative western blot for TSC1, IFT88, ASNS and S6RP of fraction 1 (Fr1: cytoplasmic), fraction 2 (Fr2: organelles), fraction 3 (Fr3: cilia) and total extract (TE) from mIMCD3 as in top. **i**, Fluorescence live imaging of eGFP-ASNS transiently transfected mIMCD3 cells in 0% FBS. eGFP-ASNS (green, arrows), nuclei (Hoechst, blue). Scale bar, 10 µm. Insets show magnification. Scale bar, 5 µm. **j**, Representative IF images of eGFP-ASNS transiently transfected mIMCD3 cells in 0% FBS. eGFP-ASNS (green), cilia (ARL13B, red), nuclei (DAPI, blue). Scale bar, 10 µm. Arrows indicate eGFP-ASNS at the base of cilia. Insets are magnifications (merged and single channels). Scale bar, 5 µm. **k**, Representative IF images of mNeon-ASNS stable mIMCD3 lines in HBSS (8 h). mNeon-ASNS (green), centrosomes (γ-tubulin, red), cilia (ARL13B, white), nuclei (DAPI, blue). Scale bar, 10 µm. Arrows indicate co-localization with centrosomes. Insets are magnifications (merged and single channels). Scale bar, 5 µm. Box-and-whisker plots show median and minimum to maximum; data in dot and bar plots are mean ± standard deviation. Average plot is mean ± standard error of the mean. Statistical analysis: one-way ANOVA, followed by Tukey’s (**a**, **d**, **e**, **g**) or Bonferroni’s (**c**) multiple comparisons test; NS, not significant, ****P < 0.0001. *n* reported in brackets. Additional replicate experiments in **d**, **e** and **h** are shown in Supplementary Fig. 7. Source data

Of interest, among the most prominent alterations that we observed in cilia-deficient cells were glutamine and asparagine, which were reduced in mutant cells (Fig. 4a). We thus investigated whether the enzyme asparagine synthetase (ASNS)^9,31^ could mediate the anaplerotic usage of glutamine and in so doing regulate the ciliary response. Indeed, silencing *Asns* induced the expected elongation of cilia at baseline and impaired the shortening of cilia upon glutamine replenishment at 8 and 24 h (Fig. 4b,c and Extended Data Fig. 6e,f). However, neither supplementation with asparagine, nor treatment of cells with asparaginase, which degrades extracellular asparagine, had an effect on ciliary length indicating that the ciliary response to glutamine does not depend on asparagine (Fig. 4d and Extended Data Fig. 6g). Conversely, silencing of *Asns* greatly dampened glutamine-driven mitochondrial respiration in both MEFs and mIMCD3 cells exposed to nutrient deprivation (Fig. 4e and Extended Data Fig. 6h), indicating that under metabolic stress conditions ASNS supports glutamate generation from glutamine to sustain mitochondrial activity via anaplerosis (Fig. 4e). Importantly, under these conditions, the silencing of *Asns* had no effect on the capability of glutamine to activate mTORC1 (Fig. 4b), further excluding a role for this kinase complex axis in the process.

In line with these data, real-time quantitative reverse transcription-polymerase chain reaction (qRT-PCR) revealed a reduced expression of ASNS in the cilia-deficient MEF^*Ift88*^ and mIMCD^*Ift88*^ as compared with controls (Extended Data Fig. 6i). ASNS uses glutamine to transamidate aspartate, thus generating asparagine and glutamate as products^31^ (Fig. 4f). Stable isotope tracing using nitrogen-labelled glutamine (^15^N_2_-glutamine) followed by LC–MS analysis, confirmed that MEF^*Ift88*^ cells showed significant reduction in glutamine uptake and a significant reduction in the release of nitrogen-labelled asparagine (m + 1) as compared with controls (Fig. 4g), supporting a reduced activity of the enzyme ASNS in cilia-deficient mutants.

Our data prompted us to ask what could be the interconnection between ASNS and primary cilia. A previous screening suggested that *Asns* could regulate ciliary length^32^, while ASNS was described as a possible component of the ciliary proteome^33^. However, none of these studies confirmed or validated these findings. To test whether ASNS could indeed be a ciliary protein, we performed an ultracentrifugation-based purification protocol to enrich for ciliary proteins^34,35^ (Fig. 4h). The results confirmed that ASNS could be found enriched in the ciliary fraction in addition to its expected cytosolic expression. Notably, two other cytosolic proteins, S6RP and TSC1, were enriched only in the cytosolic fraction, while the cilia-resident protein IFT88 was mostly enriched in cilia, demonstrating the validity of the fractionation study (Fig. 4h).

Attempts at localizing the endogenous ASNS into cilia by IF failed to identify a specific signal that could be ablated by *Asns* silencing (not shown). We thus generated multiple fluorescent protein-tagged versions of the ASNS protein. Transient transfection using a GFP-ASNS construct followed by live imaging in mIMCD3 cells revealed a diffuse cytosolic staining as expected, and a quite prominent fluorescent signal into two perinuclear and intense spots resembling centrosomes (Fig. 4i). Visualization of fixed cells to allow for counterstaining using a specific ciliary marker (ARL13B) showed that one of the two spots localized at the base of cilia (Fig. 4j). Double counterstaining of both ARL13B and the basal body and centriole marker γ-tubulin in cells stably expressing ASNS with a different fluorescent tag (mNeon-ASNS) validated the localization of ASNS at the basal body and daughter centriole (Fig. 4k). We conclude that ASNS localizes, at least in part, at the base of cilia (Fig. 4i–k).

**Extended Data Fig. 7:**
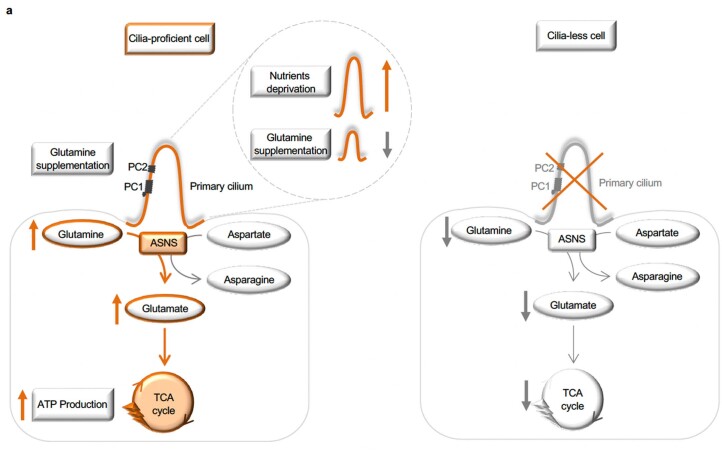
Proposed model. **a**) Schematic representation of how primary cilia sense nutrient availability and facilitate glutamine utilization through ASNS affecting the TCA cycle fuelling and, as a consequence, energy production.

In sum, our data collectively demonstrate for the first time that primary cilia respond to glutamine levels, enabling and facilitating the cellular response to glutamine during metabolic stress (glutamine anaplerosis). Our data also collectively demonstrate that ASNS is a novel centrosome/basal body protein, important to mediate the ciliary retraction in the presence of glutamine when cells are under metabolic stress, a condition associated with the required activity of ASNS to convert glutamine into glutamate to fuel the TCA cycle (Extended Data Fig. 7a).

Importantly, our data uncover a potential novel role for primary cilia in sensing nutrient availability. Future work should concentrate on identification of the precise glutamine sensory mechanism, and on the implications for physiology and pathology, particularly relevant for the ciliopathies and cancer, both areas of investigation left uncovered by our current studies.

## Methods

### Cell lines and media

MEFs are described in ref. ^36^ and MDCK cells in ref. ^37^. mIMCD3 cells were kindly provided by Dr Miriam Schmidts (Freiburg University, Germany). hRPE cells were kindly provided by Dr Nicoletta Landsberger (San Raffaele Scientific Institute, Milan, Italy). *Ift88* knockout (KO) MEFs (MEF^*Ift88*^) and mIMCD3 (mIMCD^*Ift88*^) were generated by CRISPR/Cas9 technology (see below). MEFs were stably transduced with ARL13B-GFP plasmid^18^ (Addgene, #40879) using lentiviral vectors. MEFs and MDCK cell lines were grown in 37 °C, 5% CO_2_ incubators, in high-glucose Dulbecco’s modified Eagle medium (DMEM; Thermo Fisher Scientific, #41965062). mIMCD3 cells were cultured in DMEM/F12 medium with GlutaMAX (Thermo Fisher Scientific, #31331093), supplemented with 10% foetal bovine serum (FBS), 1% penicillin–streptomycin (PenStrep; Thermo Fisher Scientific, #15070-063) and 1% sodium pyruvate (Thermo Fisher Scientific, #11360-039) or DMEM/F12 medium without l-glutamine (Thermo Fisher Scientific, #21331046) supplemented with 2.5 mM l-glutamine (Thermo Fisher Scientific, #25030-024), 10% FBS and 1% PenStrep. hRPE cells were cultured in DMEM/F12 medium without l-glutamine (Thermo Fisher Scientific, #21331046) supplemented with 10% FBS and 1% PenStrep. mNeonGreen-ASNS stable mIMCD3 cells were cultured in high-glucose, pyruvate DMEM medium (Thermo Fisher Scientific, #41966029) and Ham’s F-12 Nutrient Mix (Thermo Fisher Scientific, #21765029) (1:1), supplemented with 10% FBS, 400 µg ml^−1^ hygromycin (Sigma-Aldrich, #H0654), and 1% sodium pyruvate.

### CRISPR/Cas9 generation of *Ift88* KO cells

To generate *Ift88* KO MEFs (MEF^*Ift88*^) and mIMCD3 (mIMCD^*Ift88*^), U6gRNA-Cas9-2A-GFP plasmids (Sigma-Aldrich) carrying three distinct custom-designed guide RNA (gRNA) sequences targeting exons 5, 11 and 14 were used (gRNA#1: GATCTGATCTAAGGCCATTCGG; gRNA#2: CAAAAGACGCTTCGATCACAGG; gRNA#3: CAATGGGAAGACCGATGACAGG). For mIMCD3 cells, the most efficient guide (gRNA#1) was employed. Cells were plated on 150 mm^2^ plates the day before the transfection. Transfection was performed using Lipofectamine 3000 (Thermo Fisher Scientific, #L3000015) following the manufacturer’s instructions with 5 μg of plasmid DNA and a 1:3 DNA:Lipofectamine ratio. The CMV-Cas9-2A-RFP scrambled gRNA was used as a control. Three days after transfection, cells were FACS sorted for GFP (potential MEF^*Ift88*^ or mIMCD^*Ift88*^) or RFP (control MEFs or mIMCD3) and plated as single cells into 96-well plates. Vital clones were expanded and screened for the absence of the protein by western blot. IF staining for ARL13B confirmed the absence of cilia in *Ift88* KO MEFs or mIMCD3. Ten out of 18 vital MEF clones, and 5 out of 11 mIMCD clones were *Ift88* KO. Clones were kept in culture separately, and fresh pools of three different clones (with an equal proportion 1:3 of each single clone) for control and *Ift88* KO cells (both MEFs and mIMCD3) were used. The three individual clones were derived from three different guides. For mIMCD3 cells that are more subject to clonality problems, most experiments were also conducted with individual clones.

### In vitro treatments

To induce ciliogenesis, cells were serum starved for 24 h. For nutrient deprivation, cells were cultured in HBSS (Thermo Fisher Scientific, #14025-050) for 24 h. To analyse primary cilium response to nutrients, HBSS was supplemented with 0.2, 0.5, 1, 2 and 4 mM l-glutamine (Thermo Fisher Scientific, #25030-024), 20 mM d-(+)-glucose (Sigma-Aldrich, #G7021), 0.5 or 5 mM l-leucine (Sigma-Aldrich, #L8000), or 0.1 or 1 mM l-asparagine (Sigma-Aldrich, #A4159). For AICAR treatment, complete medium was supplemented with 1 mM AICAR (Sigma-Aldrich, #A9978) or dimethyl sulfoxide (DMSO; Sigma-Aldrich, #D2650) for either 24 h (for IF) or 4 h (for western blot analysis). For metformin treatment, complete medium was supplemented with 2 mM metformin (Sigma-Aldrich, #317240) for 4 h (for western blot analysis). For mitochondrial respiratory chain complexes inhibition, cells were treated with 1 µM oligomycin (Agilent Technologies) and 0.5 µM antimycin A/rotenone (Agilent Technologies) for 24 h. For rapamycin treatment, cells were treated with 100 nM rapamycin (LC Laboratories, #R-5000) for 24 h. For asparaginase treatment, cells were treated with 5 U ml^−1^ asparaginase (Sigma-Aldrich, #A3809) for 24 h.

### Murine models and in vivo studies

For the fasting studies, wild-type C57Bl/6N mice were starved for either 24 or 48 h. Blood was collected from mice, and serum glycaemia was detected by Glucose Hexokinase Kit (Werfen, #00018259940) following the manufacturer’s instructions. Glutamine concentration in mice serum was measured by NMR spectroscopy. Mice were perfused in phosphate-buffered saline (PBS) at 4 °C and killed at 48 h. Kidneys were collected and fixed overnight in 4% paraformaldehyde (PFA) and included in optimal cutting temperature compound (OCT). IF for primary cilium detection was performed as described below. For in vivo treatment with l-glutamine (Sigma-Aldrich, #G3126), mice were starved for 8 h and injected with l-glutamine (800 mg ml^−1^) by i.p. injection two times after 8 h and 24 h of fasting. Mice were killed at 48 h. Alternatively, a single l-glutamine i.p. injection was performed after 24 h of fasting and mice were killed at 48 h. Control mice were normally fed ad libitum, fasted for 24 h or fasted for 48 h. After sacrifice, kidneys were either fixed or liquid-nitrogen snap frozen for primary cilia IF and glutamine concentration measurement by NMR spectroscopy, respectively. For the kinetics of circulating glutamine concentration, mice were fasted for 24 h, injected with i.p. l-glutamine (800 mg ml^−1^), blood samples collected by retro-orbital withdrawal before fasting, after 24 h of fasting and after 30 min, 2 h, 6 h and 48 h after i.p. injection of glutamine, serum prepared and analysed by NMR spectroscopy. For the genetic ablation of *Opa1* in the kidney, *Opa1*^*flox/flox*^ mice (kindly provided by Dr Luca Scorrano, VIMM, Padua, Italy) and *KspCre* mice (kindly provided by Dr Peter Igarashi, University of Minnesota, Minneapolis, MN, United States) were inter-crossed to generate *Opa1*^*flox/flox*^*:KspCre* experimental mice in a pure C57BL/6N genetic background. Intra-litter *Opa1*^flox/+^:*Ksp*Cre or *Opa1*^*flox/flox*^ were used as controls. For all animal work, mice were randomized with a female-to-male ratio of 1:1. Animal care was carried out according to the institutional regulations and approved by the ethical committee for care and animal use at the San Raffaele Scientific Institute, and next approved by the Italian Ministry of Health (IACUC #921).

### Antibodies and inhibitors

For IF analysis the following antibodies were used: rabbit ARL13B (Proteintech, #17711-1-AP; 1:250), mouse acetylated α-tubulin (Sigma-Aldrich, #T6793; 1:1,000), rabbit pericentrin (Covance, #PRB-432C; 1:750) and mouse γ-tubulin (Sigma-Aldrich, #T6557; 1:5,000). Fluorochrome-conjugated secondary antibodies were the following: goat anti-rabbit AlexaFluor 488 (Thermo Fisher Scientific, #A-21441; 1:1,000), goat anti-mouse AlexaFluor 546 (Thermo Fisher Scientific, #A-11003; 1:1,000); chicken anti-mouse AlexaFluor 594 (Thermo Fisher Scientific, #A-21201; 1:1,000) and goat anti-rabbit AlexaFluor 647 (Thermo Fisher Scientific, #A-21244; 1:1,000). For nuclear staining we used 4′,6-diamidino-2-phenylindole (DAPI; Santa Cruz Biotechnology, #sc-3598; 1:5,000 or 1:10,000). For DBA-positive renal epithelial cell staining, DBA Rhodamine (Vector Laboratories, # RL-1032-2; 1:100) was used. For western blot analysis, the following antibodies were used: p-AMPK (Thr172) (Cell Signalling Technology, 2535S; 1:1,000), AMPK (Cell Signalling Technology, #2532; 1:1,000), ASNS (abcam, #ab111873), p-S6RP (s235/236) (Cell Signalling Technology, #2211s; 1:1,000), S6RP (Cell Signalling Technology, #2217; 1:1,000), Hamartin/TSC1 (Cell Signalling Technology, #4906; 1:1,000), IFT88 (Proteintech, #13967-1-AP; 1:1,000) and acetylated α-tubulin (Sigma-Aldrich, #T6793; 1:750). For housekeeping protein expression, Vinculin V284 antibody (Millipore, #05-386; 1:15,000) was used. Horseradish peroxidase (HRP)-conjugated secondary antibodies were from GE Healthcare: anti-rabbit IgG HRP linked (#934V), anti-mouse IgG HRP linked (#NA9310V) and anti-rat IgG HRP linked (#NA935V).

### IF on cells

For IF analysis, cells were plated on glass coverslips. For mIMCD3 and MDCK cell lines, coverslips were coated with fibronectin (Sigma-Aldrich, #11051407001; 1 µg ml^−1^ in PBS) before plating cells. Cells were fixed for 10 min in cold methanol or 4% PFA (Electron Microscopy Sciences, #157-4) followed by permeabilization in 0.1% Triton X-100 (Sigma-Aldrich, #T8787) in PBS. After 1 h blocking in 3% bovine serum albumin (BSA; Sigma-Aldrich, #A7906) in PBS at room temperature (RT), cells were incubated 1 h at RT or overnight at 4 °C with primary antibody diluted in 3% BSA in PBS. Cells were then incubated with secondary antibody diluted in 3% BSA in PBS for 1 h at RT and nuclei were stained with DAPI or for live imaging analysis with Hoechst 33342 (Thermo Fisher Scientific, #H3570). Glasses were then mounted with Fluorescence Mounting Medium (Dako, #S3023). Images were obtained using Zeiss Axio Observer.Z1, GE Healthcare DeltaVision Ultra and Olympus FluoVIEW 3000 RS microscopes. Quantification of both ciliary length and ciliated cells frequency was performed manually or by Accumulation and Length Phenotype Automated Cilia Analysis (ALPACA) tool using FIJI (FIJI Is Just ImageJ) software.

### IF on tissues

The frozen formalin-fixed OCT-embedded sections from control and *Opa1*^*flox/flox*^*:KspCre* kidneys samples and the relative controls were dried for 1 h at RT under chemical hood. The OCT was removed through three washings (10 min each) in PBS. The tissue sections were fixed in 4% PFA for 10 min and permeabilized with 0.2% Triton X-100 in PBS. After 1 h blocking in 3% BSA (Sigma-Aldrich, #A7906) 0.1% Triton X-100 in PBS at RT, tissue sections were incubated 1 h at RT or overnight at 4 °C with primary antibody diluted in 3% BSA in PBS. Secondary antibody was incubated with DBA in blocking solution for 1 h at RT. Nuclei were stained with DAPI (1:10,000) in PBS for 10 min at RT. Slides were mounted with Dako Fluorescence Mounting Medium. Representative images were taken using GE Healthcare DeltaVision Ultra microscope.

### COX and SDH staining

Kidneys were collected from *Opa*^*flox/flox*^*:KspCre* and control mice at postnatal day 30 (P30), weighted, and embedded directly in OCT after cardiovascular perfusion with PBS at 4 °C. Cryostat serial kidney sections (8 µm) were rehydrated with PBS, and in situ activity staining for cytochrome c oxidase (COX) and succinate dehydrogenase (SDH) enzymes was performed using COX stain (Bio-Optica; #30-30115LY) and SDH stain kits (Bio-Optica, #30-30114LY) following the manufacturer’s instructions. The slide sections were counterstained for 5 min with Hematoxylin Solution, Harris Modified solution (1:10 in distilled water; Bio-Optica) and mounted with the mounting medium. Images were acquired using Zeiss AxioImager M2m.

### TEM imaging

*Opa1*^*flox/flox*^*:KspCre* mice at P2 were weighted, and fixed for 24 h at 4 °C with 4% PFA and 2.5% glutaraldehyde in 125 mM cacodylate buffer. Kidneys were collected and post-fixed for 1 h with 2% OsO_4_ in 125 mM cacodylate buffer, washed, and embedded in Epon. Conventional thin sections (60 nm) were collected on uncoated grids, stained with uranyl and lead citrate. Imaging was performed using Zeiss Leo912 80kv Transmission Electron Microscope.

### Western blot analysis

For western blot analysis, cells or kidneys were lysed in lysis buffer solution of 150 mM NaCl (Sigma-Aldrich, #s9625), 20 mM Na_2_HPO_4_ (BDH, #10494 L)/NaH_2_PO_4_ (BDH, #102455 S), 10% glycerol (Sigma-Aldrich, #G7757), 1% Triton X-100 (pH 7.2), complete protease inhibitor cocktail (Roche, #11836145001) and phosphatase inhibitors (1 mM final concentration of glycerophosphate (Sigma-Aldrich, #G9891), sodium orthovanadate (Sigma-Aldrich, #S6508) and sodium fluoride (Sigma-Aldrich, #S6521)). Total lysates were then quantified with Bio-Rad Protein Assay Dye reagent (Bio-Rad Laboratories, #500-0006), and Laemmli buffer at a final concentration of 2× was added to the samples. Proteins were next resolved in 4–12% Tris–glycine gradient gels (Life Technologies, #NP0335BOX) and then transferred onto Immobilon-P polyvinylidene fluoride membranes (Millipore, #IPVH00010). Membranes were blocked with 5% milk in Tris-buffered saline, Tween 20 (Sigma-Aldrich, #P1379) (TBS-T). All the primary antibodies for western blot analysis were diluted in 3% BSA in TBS-T. HRP-conjugated secondary antibodies were diluted 1:10,000 in 5% milk, TBS-T, and detection was performed with ECL (GE Healthcare, #RPN2106) alone or supplied with 10% SuperSignal West Femto (Thermo Fisher Scientific, #34095) when necessary.

### Real-time PCR analysis

Total RNA was isolated from cells or kidneys using the RNAspin Mini kit (GE Healthcare, #25-0500-72). Complementary DNA was obtained by reverse transcription of extracted RNA using Oligo(dT)_15_ primers (Promega, #C1101) or Random Primers (Promega, #C1181) and ImProm-II Reverse Transcriptase (Promega, #A3802). Quantitative real-time PCR analysis was performed on technical duplicates using iTaq Univer SYBR Green (Bio-Rad Laboratories, #1725125) on CFX96 Touch Real-Time PCR Detection System (Bio-Rad Laboratories). Primer sequences for qRT–PCR are reported below:

*mHprt* fw5′-TTATGTCCCCCGTTGACTGA-3′

*mHprt* rev5′-ACATTGTGGCCCTCTGTGTG-3′

*mAsns* fw5′-GGTTTTCTCGATGCCTCCTT-3′

*mAsns* rev5′-TGTGGCTCTGTTACAATGGTG-3′

### *Asns* transient knockdown

For transient *Asns* gene silencing in mIMCD3 cells, 20 nM *Asns* siRNA (Thermo Fisher Scientific, #AM16704/188316) and scramble (Scr) siNegative n. 1 (Thermo Fisher Scientific, #AM4611) or n. 2 (Thermo Fisher Scientific, #AM4613) for controls were used following the manufacturer’s instructions. For siRNA transfection, cells were seeded on a 100 mm^2^ plate. The transfections were performed two times over 2 days using Lipofectamine 3000 or Lipofectamine RNAiMAX Transfection Reagent (Thermo Fisher Scientific, #13778150) following the manufacturer’s protocol. After transfections, cells were plated for total RNA extraction, protein extraction, IF and Seahorse analysis.

### eGFP-ASNS transient transfection

The plasmids for expression of ASNS (p-ASNS) and N-terminally tagged eGFP-ASNS recombinant protein (p-eGFP-ASNS) were generated by Genscript Biotech Corp. For transient transfection of p-eGFP-ASNS, mIMCD3 cells were plated in 12-well plates. Transfection was performed using Lipofectamine 3000 (Thermo Fisher Scientific, #L3000015) following the manufacturer’s instructions. One microgram of plasmid DNA per well with 1:2 DNA:Lipofectamine ratio was used. After transfections, cells were used for IF staining.

### Cloning of ASNS plasmids

ASNS expression vector (p-ASNS) was used as a PCR template to generate an Entry clone for the Gateway cloning system (Thermo Fisher Scientific). The Entry clone was sequence verified and used to create an N-terminal mNeonGreen-ASNS fusion using a pgLAP1-mNeonGreen destination plasmid (DEST).

### Generation of mIMCD3 cells stably expressing mNeonGreen-ASNS

The stably expressing mNeonGreen-ASNS cell line was generated using Flp-In mIMCD3 (a kind donation by Dr M. Nachury). Cells were plated in triplicate on six-well plates at 15% confluency in DMEM/F12 medium, supplemented with 10% FBS and 1% sodium. The next day, reaching 70% confluency, cells were co-transfected with 1 µg ml^−1^ of pOG44 vector (ThermoFisher, #V600520) and DEST vector using Lipofectamine 2000 (ThermoFisher, #11668019) in a 1:2 dilution (DNA:Lipofectamine) according to the manufacturer’s instructions. pOG44 is the Flp-Recombinase Expression Vector, which allows for substitution of the FRT cassette with mNeonGreen-ASNS. After 48 h from transfection, cells were grown in selection medium with 400 µg ml^−1^ hygromycin. Medium with hygromycin was refreshed every 2–3 days for 1.5 weeks. IF and western blot confirmed protein expression of construct.

### Seahorse metabolic flux analysis

The Mito Stress Test (Agilent Technologies) was performed by seeding 20,000 cells per well in 96-well Seahorse cell culture microplates and incubating them in a 5% CO_2_ incubator at 37 °C overnight. Then, culture medium was changed with pH adjusted (pH 7.4 ± 0.1) with 2 mM HEPES bicarbonate-free HBSS, and pH adjusted with 2 mM HEPES HBSS supplemented either with 20 mM d-(+)-glucose or 4 mM l-glutamine for 4, 16 and 24 h. The plate was incubated at 37 °C for 1 h in a non-CO_2_ incubator before starting the assay. OCR was measured using the Seahorse XF Mito Stress Test Kit (Agilent Technologies, #103015-100) on an XFe96 Analyzer (Agilent Technologies) following the manufacturer’s instructions. Briefly, cells were sequentially injected with 1 μM oligomycin, 1.5 μM carbonyl cyanide-4 (trifluoromethoxy) phenylhydrazone (FCCP) and 0.5 μM antimycin A/rotenone. For measuring OCR in response to acute injection of nutrients, Mito Stress Test was performed on cells treated for 4 h (or 16 h in Supplementary Information) with pH adjusted with 2 mM HEPES HBSS and acutely injected with 20 mM d-(+)-glucose or 4 mM l-glutamine. For measuring OCR of *Ift88* control and KO MEFs and mIMCD3 in nutrient-rich condition cells either with or without FBS cells were cultured for 24 h in DMEM or DMEM/F12 ± 10% FBS. Then, culture medium was changed with Seahorse XF DMEM medium (Agilent Technologies, #103575-100) and the plate was incubated at 37 °C for 1 h in a non-CO_2_ incubator before starting the assay. Cell numbers were normalized using CyQuant Cell Proliferation Assay (Thermo Fisher Scientific, #C35011). All the analyses were performed with the Agilent Seahorse Wave software (Agilent Technologies).

### Cilia enrichment

Cells were seeded on a 150 mm^2^ dish at 90% confluence. Cells were starved for 24 h or 48 h in DMEM/F12 with GlutaMAX supplemented with 0% FBS and 1% PenStrep. After 10 min incubation in PBS supplemented with 1 mM of EDTA cells were scraped, centrifuged at 200*g* for 5 min and washed with HBS (25 nM HEPES, 137 mM NaCl, 5 mM KCl, 0.7 mM Na_2_HPO_4_·2H_2_O and 6 mM d-(+)-glucose pH 7.05). Pellet was resuspended in 1 ml of Deciliation solution (20 mM HEPES pH 7, 112 mM NaCl, 3.4 mM KCl, 10 mM CaCl_2_, 2.4 mM NaHCO_3_ and 20% ethanol) with 10 µg ml^−1^ cytochalasin D and 1% protease inhibitor cocktail for 15 min at 4 °C rotating. After a centrifugation at 1,000*g* for 5 min at 4 °C, sequential centrifugations for 30 min at 4 °C were performed. The first centrifugation was performed at 2,000*g* to collect the first cytoplasmic fraction. The second centrifugation was performed at 10,000*g* to collect the second organelles fraction. The third centrifugation was performed at 16,000*g* to collect the third cilia-enriched fraction.

### NMR exometabolome analysis

*Ift88* control and KO MEFs were seeded in 100 mm^2^ plates in complete medium. Then, the culture medium was replaced overnight with high-glucose DMEM medium supplemented with 0.5% FBS and 1% PenStrep. The day after, the culture medium was replaced with high-glucose DMEM medium supplemented with 0% FBS and 1% PenStrep for 24 h. A blank control with non-conditioned medium was used to calculate the metabolite relative quantification and consumption. For NMR analysis of the extracellular medium, 530 μl of cell culture medium were mixed with 60 μl of deuterated sodium phosphate (Na_3_PO_4_) solution containing 4,4-dimethyl-4-silapentane-1-sulfonic acid, as a chemical shift reference for proton dimension, and 10 μl of 1.2% NaN_3_ water solution. The final sample volume was 600 μl and contained 50 mM sodium phosphate (Na_3_PO_4_), 0.02% NaN_3_ and 50 μM 4,4-dimethyl-4-silapentane-1-sulfonic acid. NMR spectra were recorded at 298K on a Bruker Avance 600 Ultra Shield TM Plus 600 MHz spectrometer equipped with triple resonance cryoprobe, pulsed field gradients and refrigerated autosampler (SampleJet). Samples were stored at 4 °C until data collection. 1D ^1^H NMR spectra (noesypr1d) were recorded with an acquisition time of 3 s, 128 transients and a relaxation delay of 6 s. Spectral window was set to 14 ppm. 1D ^1^H NMR spectra were typically processed with zero filling to 128k points, and apodized with an unshifted Gaussian and a 1 Hz line broadening exponential using Mnova 14.1 (MestreLab Research S.L, Santiago de Compostela). Metabolites were identified and quantified using Chenomx NMR Suite 8.6 (Chenomx). Relative quantification (Rel.Quant.) was calculated using the following equation:

$${\mathrm{Rel.Quant.}} = \frac{{\left[ {{\mathrm{Meta}}} \right]x - \left[ {{\mathrm{Meta}}} \right]{\mathrm{mo}}}}{{\left[ {{\mathrm{Meta}}} \right]{\mathrm{mo}}}}$$

where [Meta]*x* represents the concentration of metabolites in the medium conditioned by *Ift88* control and KO MEFs; [Meta]mo represents the concentration of metabolites in the non-conditioned medium. Negative values corresponded to metabolites up-taken from the medium, whereas positive values corresponded to metabolites released in the medium.

Metabolite concentration, expressed in mM, was normalized for the total number of cells. The reported concentration of the metabolites is expressed as nM/number of cells.

Glucose or glutamine consumption was calculated using the following equation:

$${\mathrm{Consumption}} = \frac{{\left[ {{\mathrm{Meta}}} \right]{\mathrm{mo}} - \left[ {{\mathrm{Meta}}} \right]x}}{{{\mathrm{finalnumberofcells}}}}$$

where [Meta]*x* is the concentration of glucose or glutamine in the medium conditioned by *Ift88* control and KO MEFs, and [Meta]mo represents the concentration of metabolites in the non-conditioned medium.

Principal component analysis was performed using the 25 identified and quantified metabolites in the exometabolome samples.

Raw data of NMR exometabolome analysis are reported in Extended Data Table 1.

### Targeted metabolomic analysis in *Ift88* KO MEFs

The unlabelled targeted metabolomics in *Ift88* control and KO MEFs were performed by plating the cells in five biological replicates, and culturing for 24 h with either HBSS or HBSS supplemented with 4 mM glutamine. Cell pellets were extracted with 1 ml extraction solution, that is, methanol for highly pure liquid chromatography (Sigma-Aldrich): acetonitrile gradient grade for liquid chromatography (Merck): ultrapure water (Sigma-Aldrich), 50:30:20 with 100 ng ml^−1^ of HEPES (Sigma-Aldrich) per million cells. Extracellular metabolites were extracted with 750 μl of extraction solution to 50 μl cell culture medium (spun).

Samples were incubated at 4 °C for 15 min, centrifuged at 13,000 r.p.m, and the supernatant transferred into autosampler vials was stored at −80 °C. Separation of metabolites by LC–MS chromatography was performed using a Millipore Sequant ZIC-pHILIC analytical column (5 µm, 2.1 × 150 mm) equipped with a 2.1 × 20 mm guard column (both 5 mm particle size) and a binary solvent system. Solvent A was: 20 mM ammonium carbonate and 0.05% ammonium hydroxide; solvent B was acetonitrile. The column oven was kept at 40 °C and the autosampler tray at 4 °C. The gradient for chromatographic separation ran at a flow rate of 0.200 ml min^−1^: 0–2 min: 80% B; 2–17 min: linear gradient from 80% B to 20% B; 17–17.1 min: linear gradient from 20% B to 80% B; 17.1–22.5 min: hold at 80% B. Next, samples were randomized and analysed by LC–MS injecting a volume of 5 µl. An equal mixture of all individual samples was used to generate pooled samples next analysed interspersed at regular intervals within sample sequence as a quality control. Metabolites were measured using a Thermo Scientific Q Exactive Hybrid Quadrupole-Orbitrap Mass spectrometer (HRMS) that was coupled to a Dionex Ultimate 3000 UHPLC. The full-scan, polarity-switching mode was used to operate the mass spectrometer, using the spray voltage set to +4.5 kV/−3.5 kV. Furthermore, the heated capillary was held at 320 °C and the auxiliary gas heater at 280 °C. The sheath gas flow was set to 35 units, the auxiliary gas flow to 10 units and the sweep gas flow to 0 units. HRMS data acquisition was performed in a range of *m*/*z* = 70–900, with the resolution set at 70,000, the AGC target at 1 × 10^6^ and the maximum injection time (Max IT) at 120 ms. The identities of the metabolites was confirmed as follows: (1) using precursor ion *m*/*z* was matched within 5 p.p.m. of theoretical mass predicted by the chemical formula; (2) using a retention time of metabolites within 5% of the retention time of a purified standard that was run in identical chromatographic conditions. The review of the chromatogram and that of the peak area integration were performed using the ThermoFisher software Tracefinder 5.0. The area of the peak for each detected metabolite was normalized using the total ion count of the same sample to correct for any variations that had been introduced by sample handling and instrument analysis. All the normalized areas were used as variables for further statistical data analysis. For glutamine tracing experiments, *Ift88* control and KO MEFs were cultured in HBSS supplemented with 4 mM ^15^N_2_-glutamine (Cambridge Isotope Laboratories) for 4 h or 24 h. Cells were seeded in parallel plates and protein content was determined by the Bradford method at 0 and 24 h post medium change. The extraction of intracellular and extracellular metabolites was carried out in the same way as the unlabelled metabolomics. The theoretical masses of ^15^N-isotopologues for each metabolite were calculated and added to a library of predicted isotopologues. These masses were then searched within a 5 p.p.m. tolerance and integrated only if the peak showed less than 1% difference in retention time from the [U-^14^N] monoisotopic mass in the same chromatogram. Natural isotope abundances were corrected using the AccuCor algorithm (https://github.com/lparsons/accucor). Percentage of intracellular pool from each isotopologue was calculated respective of the control (for each metabolite).

$${\bar{m}}_x{\log}_2{\bar{m}}_{{\mathrm{HBSS}} + {\mathrm{Q}}} < {\bar{m}}_{{\mathrm{HBSS}}}{\log}_2{\bar{m}}_{{\mathrm{HBSS}} + {\mathrm{Q}}} > {\bar{m}}_{{\mathrm{HBSS}}}$$

TIC normalized data of LC–MS analysis are reported in Supplementary Data 1.

### Metabolite analysis

We applied fold-change and *t*-test analysis to identify dysregulated metabolites in the different conditions. For each *P* value resulting from *t*-test, a false discovery rate (FDR) has been computed by applying the Benjamini and Hochberg procedure as in Podrini et al.^23^. Then, only metabolites having FDR <0.05 have been considered as significantly deregulated. Heat maps have been created by applying the MATLAB^38^ heatmap function with colormap represented in logarithmic scale. Volcano plots have been obtained by a homemade MATLAB function (available at https://github.com/RobertoPagliarini/PrimaryCilia).

### Metabolite set enrichment analysis

Over-representation analysis has been employed, by using MetaboAnalyst 5.0 (ref. ^39^), to identify pathways that are significantly enriched in the Kyoto Encyclopedia of Genes and Genomes database^40^ starting from an input list of metabolites. We applied the hyper-geometric test to compute a statistical significance (*P* value) for each pathway having at least three compounds captured in the input list. Hyper-geometric test scores have been computed on the basis of cumulative binominal distribution, while FDR has been obtained by applying the Benjamini and Hochberg procedure.

### Statistical analysis and replicate experiments

Differences between averages were established with Student’s *t*-test or one-way analysis of variance (ANOVA) as indicated in the figure legends; Tukey’s or Bonferroni’s post-tests were carried out for multiple comparisons. Dot plots show the individual datapoints with the respective *n* indicated in brackets. Whenever representative of multiple experiments are shown, the replicate experiments are provided in Supplementary Information. Whenever average of the average of multiple experiments is shown, this is indicated in the legend.

### Reporting summary

Further information on research design is available in the Nature Portfolio Reporting Summary linked to this article.

### Supplementary information

Supplementary Information Supplementary Figs. 1–8, methods and uncropped western blots for Supplementary Fig. 7c.

Reporting Summary

Supplementary Data 1 Raw data from LC–MS.

### Source data

Source Data Fig. 1 Statistical source data.

Source Data Fig. 2 Statistical source data.

Source Data Fig. 3 Statistical source data.

Source Data Fig. 4 Statistical source data.

Source Data Extended Data Fig. 1 Statistical source data.

Source Data Extended Data Fig. 2 Statistical source data.

Source Data Extended Data Fig. 3 Statistical source data.

Source Data Extended Data Fig. 4 Statistical source data.

Source Data Extended Data Fig. 5 Statistical source data.

Source Data Extended Data Fig. 6 Statistical source data.

Uncropped Western Blots Uncropped western blots for Fig. 4b,h and Extended Data Figs. 1a,d, 3c and 6a,c.

## Figures and Tables

**Extended Data Table 1:**
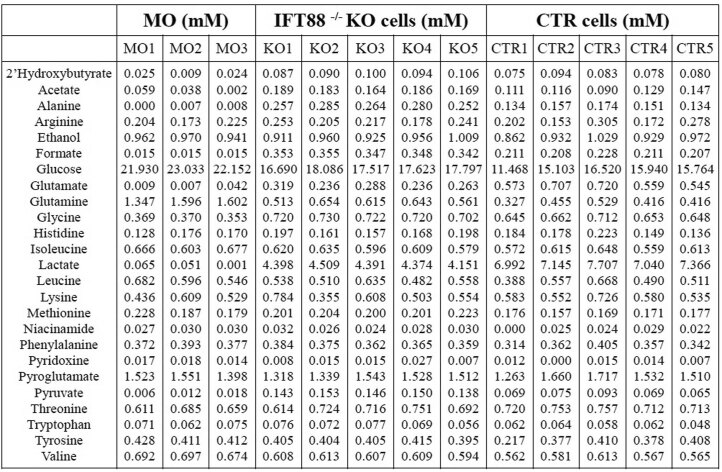
List of NMR metabolites

List of identified and quantified metabolites by NMR. For each metabolite, concentration is expressed as mM for unconditioned medium (MO) and for media conditioned by *Ift88* KO and CTR cells. For MO, three replicates have been used. For KO and CTR, five replicates have been used.

**Extended Data Table 2:**
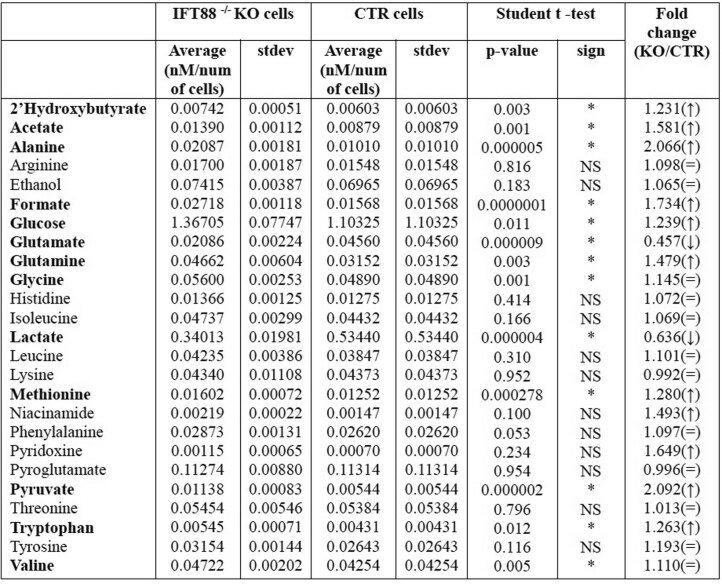
Univariate analysis NMR metabolites

List of identified and quantified metabolites by NMR and the corresponding univariate analysis. For each metabolite, average and standard deviation calculated for KO and CTR samples. Fold change (FC), considered as the ratio between the mean value of the two groups KO and CTR, metabolites with a FC >1.2 (↑) indicates an increase of metabolite, FC <0.7 (↓) indicates a decrease, 0.7 < FC < 1.2 is not a variation of metabolite (=). *P* value is calculated using Student’s two-tailed *t*-test (fourth column). Statistically significant differences in metabolites in KO versus CTR are expressed in bold and asterisk. NS, not significant; *P < 0.05.

## Data Availability

Source data are provided with this paper. All raw data related to the studies shown in figures and extended data figures. The original TIFF files used for generating the raw data relative to quantification of ciliary length are available in Figshare (10.6084/m9.figshare.21922299).
