# Supplementary material for: Primary cilia sense glutamine availability and respond via asparagine synthetase
Source: Nat Metab. 2023 Mar 6;5(3):385–97. doi: 10.1038/s42255-023-00754-6 (PMC10042734; doi:10.1038/s42255-023-00754-6)
Supplement: Supplementary file 1 — Supplementary Figs. 1–8, methods and uncropped western blots for Supplementary Fig. 7c. [file 42255_2023_754_MOESM1_ESM.pdf]

nature metabolism

Letter

https://doi.org/10.1038/s42255-023-00754-6

Primary cilia sense glutamine availability and respond via asparagine synthetase

In the format provided by the authors and unedited

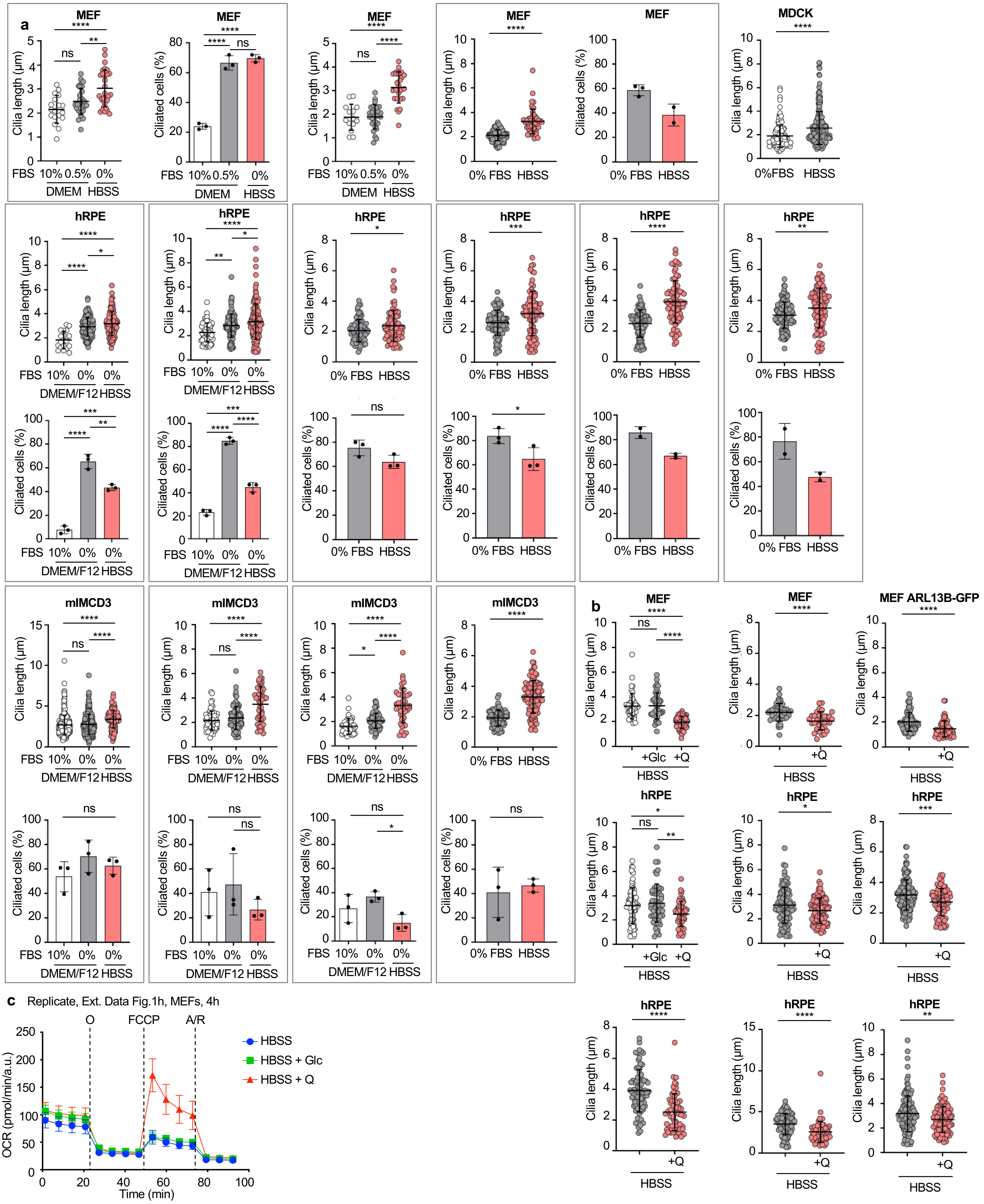

Supplementary Fig. S1 (Referred to Fig.1).

a) Quantification of cilia length and percentage (%) of ciliated cells in additional replicate experiments in the indicated cell lines in the indicated conditions referred to Fig. 1b. **b)** Quantification of cilia length and percentage (%) of ciliated cells in additional replicate experiments in the indicated cell lines in the indicated conditions referred to Fig. 1f. **c)** Analysis of OCR measurement in additional replicate experiment in MEF cells cultured in HBSS for 4h in basal condition and after sequential addition of oligomycin (O), FCCP, and antimycin A/rotenone (A/R) referred to Fig. 1h. Data in dot and bar plots are mean ± SD. Statistical analysis: Student's unpaired two-tailed *t*-test or one-way ANOVA, followed by Tukey's multiple comparisons test; ns: not significant, *p < 0.05, **p < 0.01, ***p < 0.001, ****p < 0.0001.

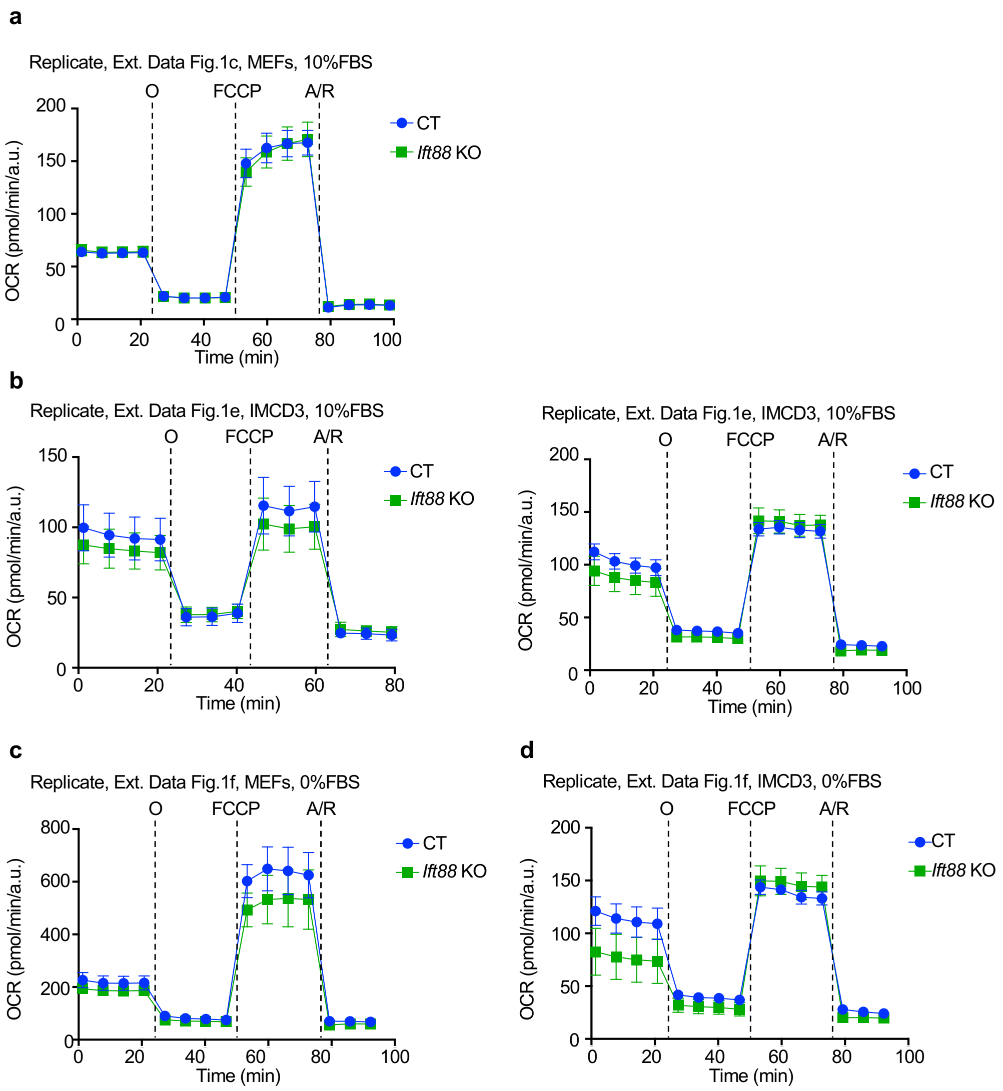

Supplementary Fig. S2 (Referred to Extended Data Fig.1).

a) Analysis of OCR measurement in additional replicate experiment in MEF^{Ift88} and MEF^{Ctrl} cultured in DMEM + 10% FBS in basal condition and after sequential addition of oligomycin (O), FCCP, and antimycin A/rotenone (A/R) referred to Extended Data Fig. 1c. **b**) Analysis of OCR measurement in additional replicate experiments in mIMCD3^{Ift88} and mIMCD3^{Ctrl} cultured in DMEM/F12 + 10% FBS in basal condition and after sequential addition of O, FCCP, and A/R referred to Extended Data Fig. 1e. **c**) Analysis of OCR measurement in additional replicate experiment in MEF^{Ift88} and MEF^{Ctrl} cultured in DMEM + 0% FBS in basal condition and after sequential addition of O, FCCP, and A/R referred to Extended Data Fig. 1f (Left). **d**) Analysis of OCR measurement in additional replicate experiment in mIMCD3^{Ift88} and mIMCD3^{Ctrl} cultured in DMEM/F12 + 0% FBS in basal condition and after sequential addition of O, FCCP, and A/R referred to Extended Data Fig. 1f (Right). Data in dot and bar plots are mean ± SD.

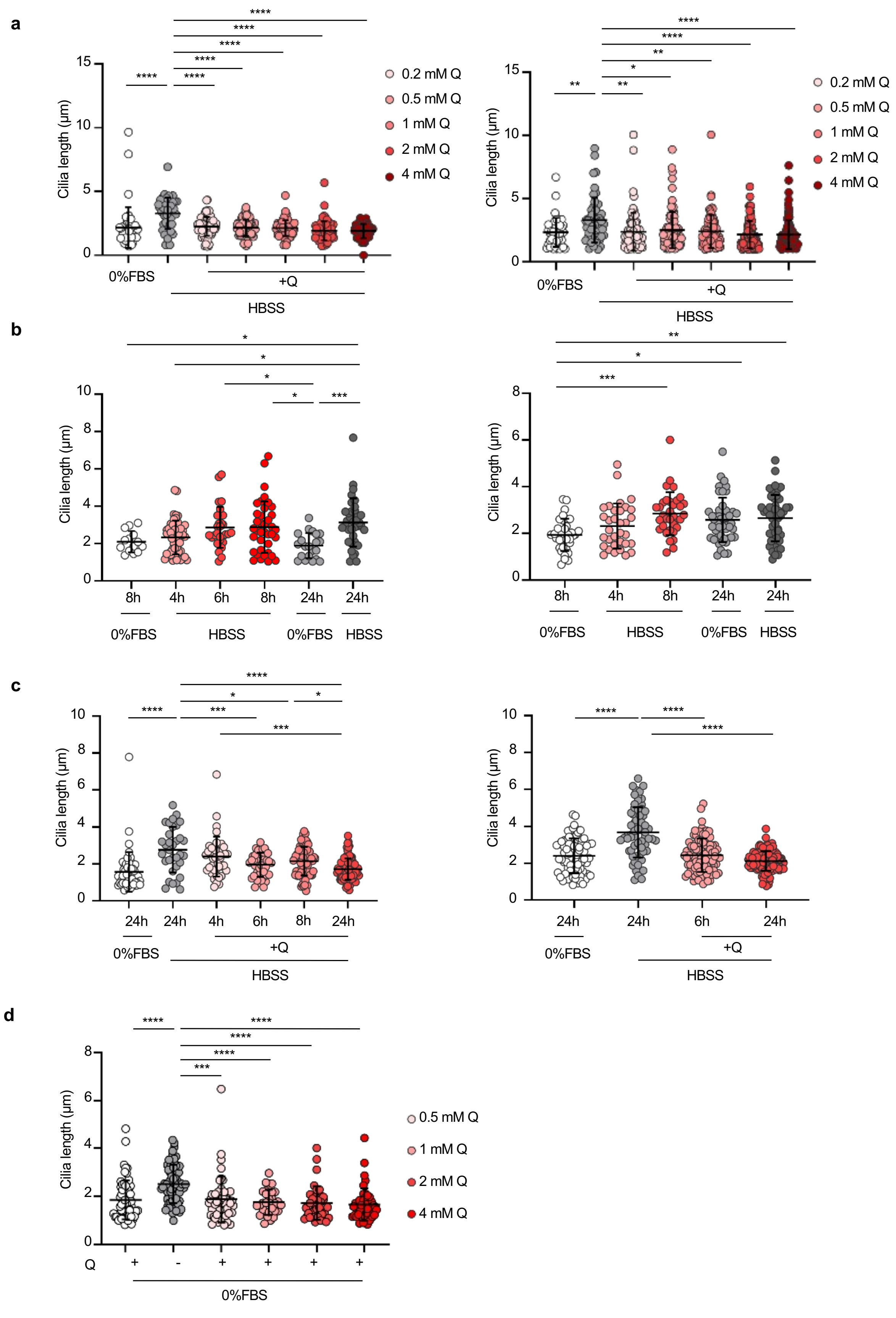

Supplementary Fig. S3 (Referred to Fig.2).
a) Quantification of cilia length in additional replicate experiments in mIMCD3 after 24 hours culture in DMEM/F12 + 0% FBS and HBSS ± L-Glutamine (Q) (0.2, 0.5, 1, 2, 4 mM) referred to Fig. 2a. **b**) Quantification of cilia length in additional replicate experiments in mIMCD3 cultured for either 8 or 24 hours in DMEM/F12 + 0% FBS and for 4, 6, 8, 24 hours in HBSS referred to Fig. 2b. **c**) Quantification of cilia length in additional replicate experiments in mIMCD3 cultured for 24 hours in either DMEM/F12 + 0% FBS or HBSS and replenished with Q for 4, 6, 24 hours referred to Fig. 2c. **d**) Quantification of cilia length in additional replicate experiment in mIMCD3 after 24 hours culture in DMEM/F12 + 0% FBS ± Q (0.5, 1, 2, 4 mM) referred to Fig. 2d. Data in dot and bar plots are mean ± SD. Statistical analysis: one-way ANOVA, followed by Tukey's multiple comparisons test; ns: not significant, *$p < 0.05$, **$p < 0.01$, ***$p < 0.001$, ****$p < 0.0001$.

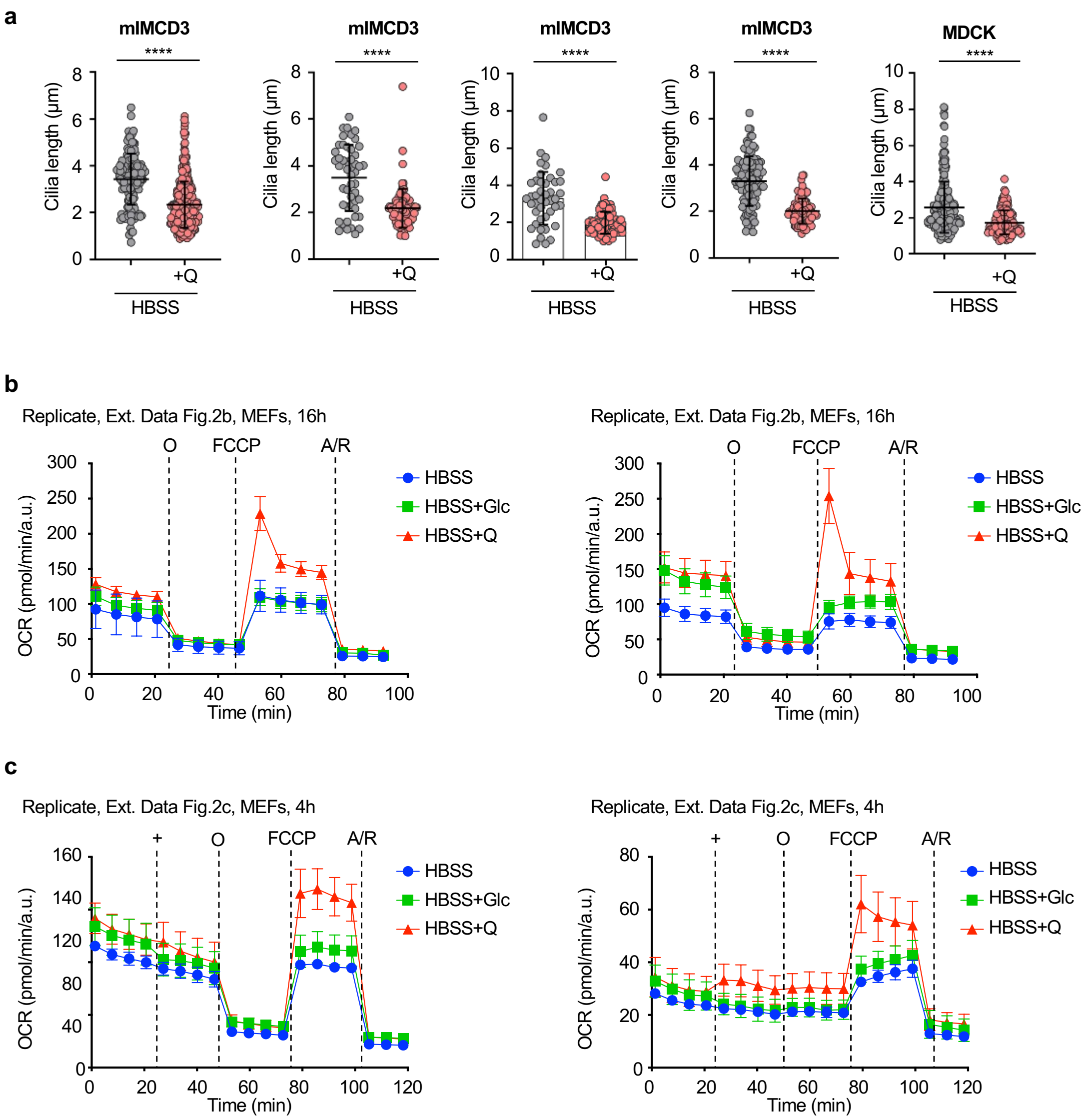

Supplementary Fig. S4 (Referred to Extended Data Fig.2).

a) Quantification of cilia length in additional replicate experiments in mIMCD3 and MDCK, after 24 hours culture in HBSS ± L-Glutamine (Q) (4 mM) referred to Extended Data Fig. 2a. **b**) Analysis of OCR measurement in additional replicate experiments in MEF after 16 hours culture in either HBSS (blue) or HBSS + D-(+)-Glucose (Glc) (20 mM) (green) or Q (4 mM) (red) in basal condition and after sequential addition of O, FCCP, and A/R referred to Extended Data Fig. 2b. **c**) Analysis of OCR measurement in additional replicate experiments in MEF after 4 hours culture in HBSS (blue) followed by acute injection (+) of either Glc (green) or Q (red) and after sequential addition of O, FCCP and A/R referred to Extended Data Fig. 2c. Data in dot and bar plots are mean ± SD. Statistical analysis: Student's unpaired two-tailed t-test; ****$p < 0.0001$.

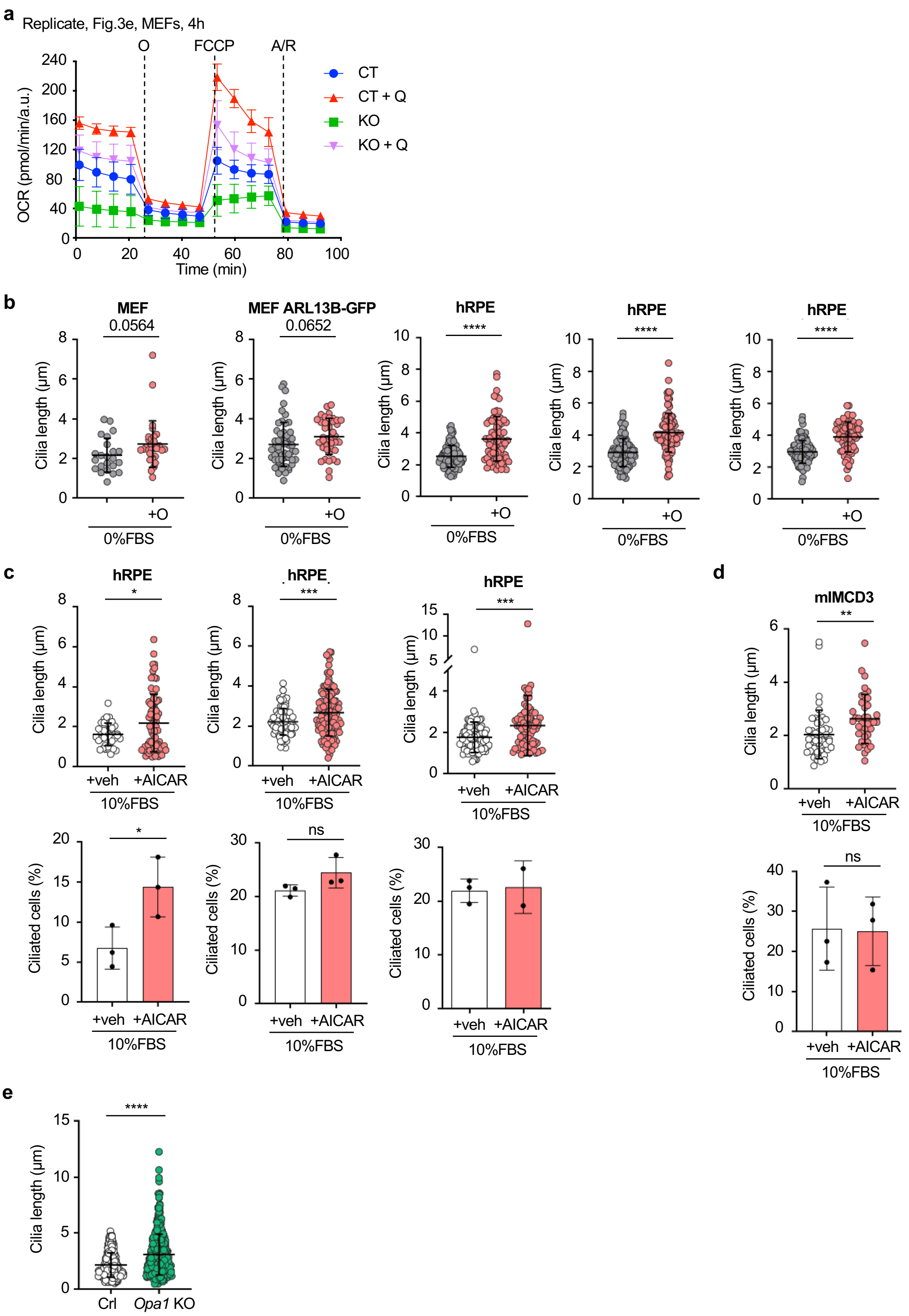

Supplementary Fig. S5 (Referred to Fig. 3 and Extended Data Fig. 3,4).

a) Analysis of OCR measurement of additional replicate experiment in MEF^{Ift88} and MEF^{Ctrl} after 4 hours culture in either HBSS (CT: blue, KO: green) or HBSS + L-Glutamine (Q) (4 mM) (CT: red, KO: purple) in basal condition and after sequential addition of oligomycin (O), FCCP, and antimycin A/rotenone (A/R) referred to Fig. 3e. **b**) Quantification of cilia length in additional replicate experiments in MEF and hRPE after 24 hours culture in DMEM or DMEM/F12 + 0% FBS ± O referred to Extended Data Fig. 3b. **c**) Quantification of cilia length and % of ciliated cells in additional replicate experiments in hRPE after 24 hours culture in the indicated conditions referred to Extended Data Fig. 3e (Left). **d**) Quantification of cilia length and % of ciliated cells in additional replicate experiments in mIMCD3 after 24 hours culture in the indicated conditions referred to Extended Data Fig. 3e (Right). **e**) Quantification of cilia length in additional replicate experiment of DBA+ tubular cells in kidney sections of Crl and *Opa1* KO mice at P30 referred to Extended Data Fig. 4f. Data in dot and bar plots are mean ± SD. Statistical analysis: Student's unpaired two-tailed t-test; ns: not significant, *p < 0.05, **p < 0.01, ***p < 0.001, ****p < 0.0001.

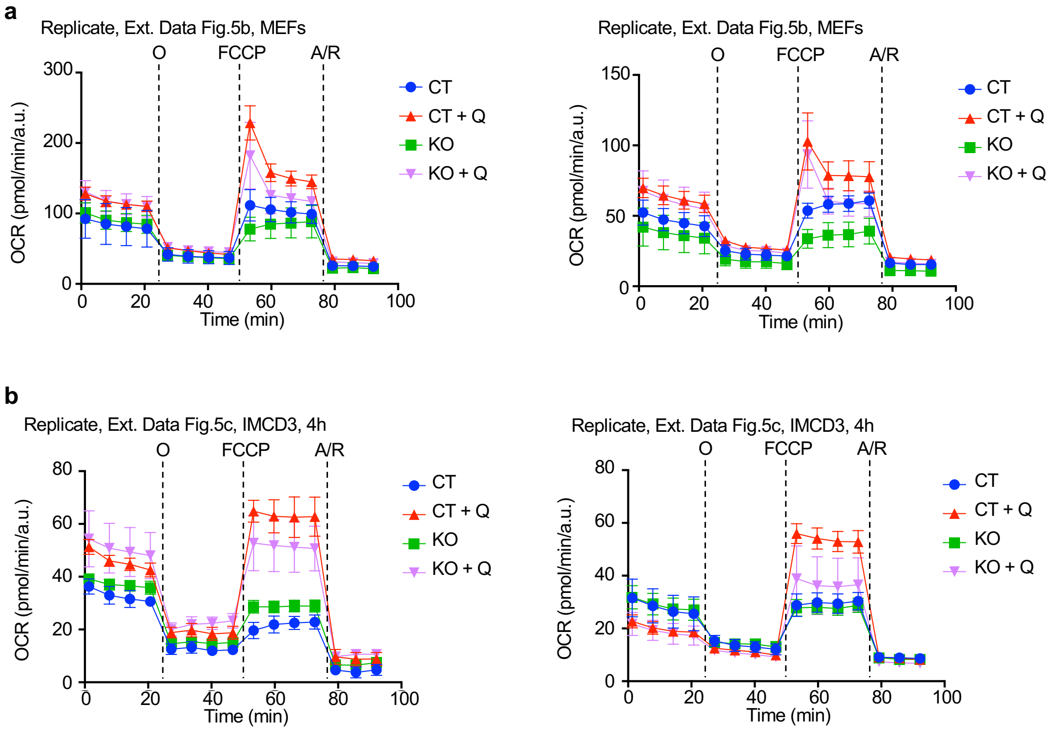

Supplementary Fig. S6 (Referred to Extended Data Fig. 5).

a) Analysis of OCR measurement in additional replicate experiments in MEF^{Ift88} and MEF^{Ctrl} after 16-24 hours culture in either HBSS (CT: blue, KO: green) or HBSS + L-Glutamine (Q) (4 mM) (CT: red, KO: purple) in basal condition and after sequential addition of oligomycin (O), FCCP, and antimycin A/rotenone (A/R) referred to Extended Data Fig. 5b. **b**) Analysis of OCR measurement in additional replicate experiments in mIMCD3^{Ift88} and mIMCD3^{Ctrl} cells after 4 hours culture in either HBSS (CT: blue, KO: green) or HBSS + Q (4 mM) (CT: red, KO: purple), in basal condition and after sequential addition of O, FCCP, and A/R referred to Extended Data Fig. 5c.

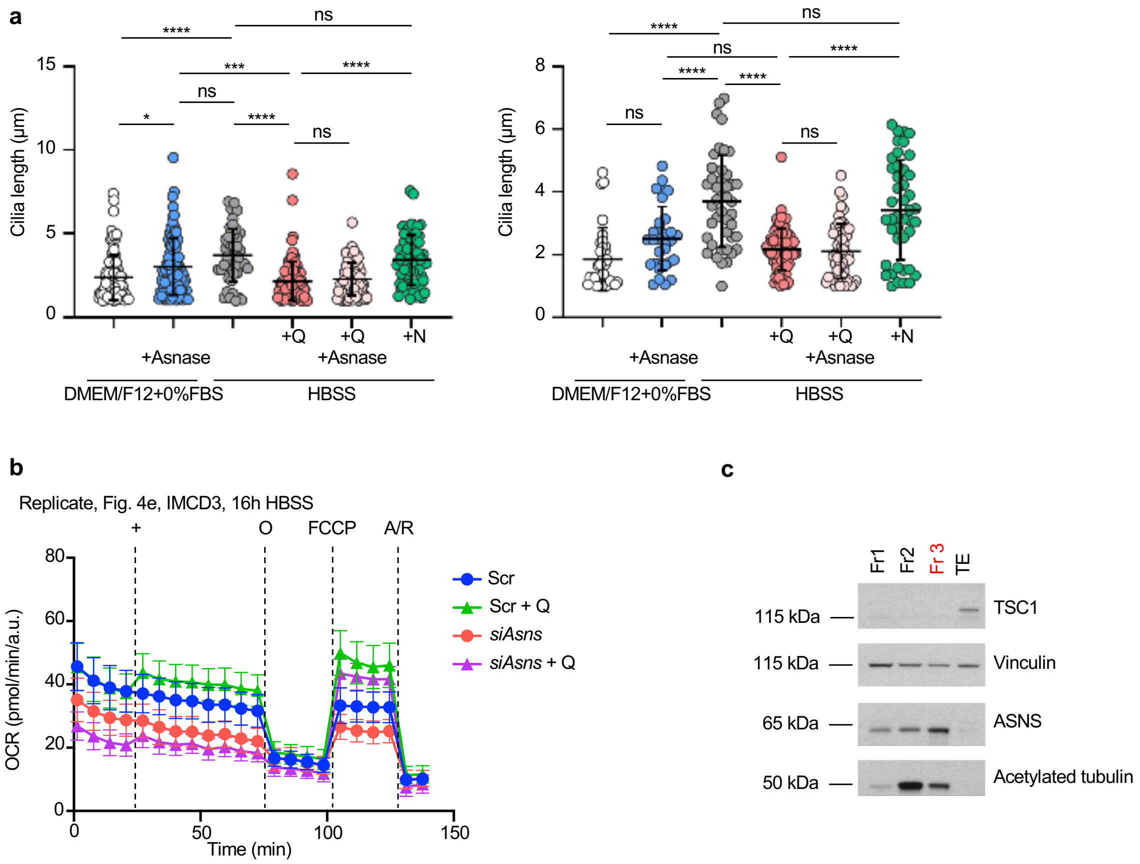

Supplementary Fig. S7 (Referred to Fig. 4).

a) Quantification of cilia length of additional replicate experiments in mIMCD3 after 24 hours culture in either DMEM/F12 + 0% FBS ± Asnase (5 U/mL), HBSS ± L-Glutamine (Q), or Q and Asnase, or L-Asparagine (N) (0.1 mM) referred to Fig. 4d. **b**) Analysis of OCR measurement of additional replicate experiment in mIMCD3 transiently knocked down for *Asns* (si*Asns*) compared to control (Scr) after 16 hours culture in HBSS (Scr: blue, si*Asns*: red) followed by acute injection (+) of Q (Scr: green, si*Asns*: purple), in basal condition and after sequential addition of oligomycin (O), FCCP, and antimycin A/rotenone (A/R) referred to Fig. 4e. **c**) Additional replicate experiment of western blot for TSC1, Vinculin, ASNS, Acetylated tubulin of Fraction 1 (Fr1: cytoplasmic), Fraction 2 (Fr2: organelles), Fraction 3 (Fr3: cilia enriched) and total extract (TE) from scramble and si*Asns* mIMCD3 in the indicated conditions referred to Fig. 4h. Statistical analysis: Student's unpaired two-tailed *t*-test or one-way ANOVA, followed by Tukey’s multiple comparisons test; ns: not significant, *$p < 0.05$, ***$p < 0.001$, ****$p < 0.0001$.

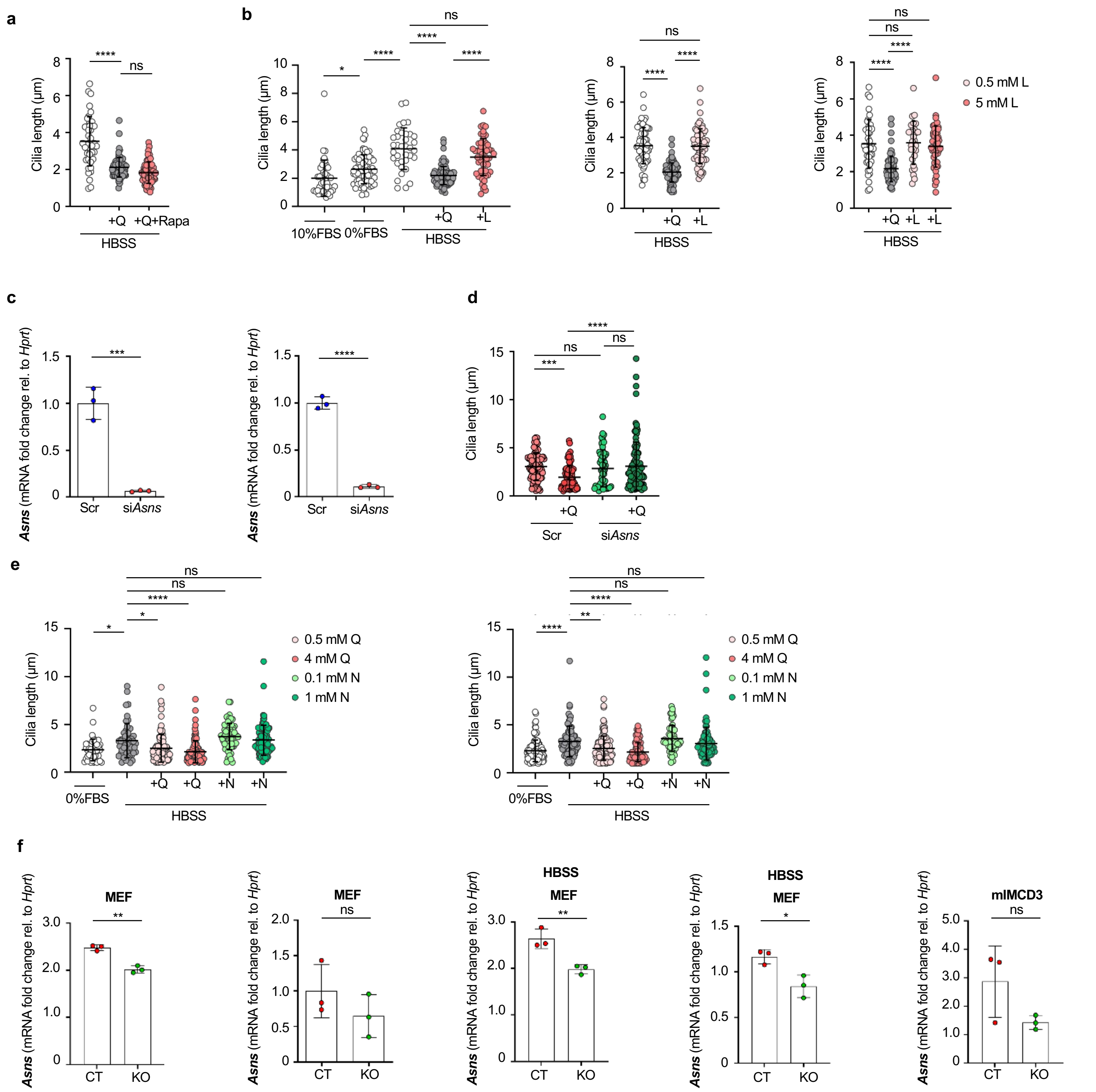

Supplementary Fig. S8 (Referred to Extended Data Fig. 4).

a) Quantification of cilia length in additional replicate experiment in mIMCD3 cells after 24 hours culture in either HBSS ± L-Glutamine (Q) or HBSS + Q + Rapamycin (Rapa) (100 nM) referred to Extended Data Fig. 6b. **b**) Quantification of cilia length in additional replicate experiments in mIMCD3 cells after 24 hours culture in either HBSS ± Q or L-Leucine (L) 0.5 or 5 mM) referred to Extended Data Fig. 6d. **c**) qRT-PCR analysis of *Asns* expression in additional replicate experiments in mIMCD3 transiently knocked down for *Asns* (si*Asns*) relative to control (Scr) referred to Extended Data Fig. 6e. **d**) Quantification of cilia length in additional replicate experiment in mIMCD3 transiently knocked down for *Asns* (si*Asns*) relative to control (Scr) after 8 hours culture in HBSS or HBSS + Q (4 mM) referred to Extended Data Fig. 6f. **e**) Quantification of cilia length in additional replicate experiments in mIMCD3 after 24 hours culture in either DMEM/F12 + 0% FBS or HBSS or HBSS + Q (0.5 or 4 mM) or HBSS + L-Asparagine (N) (0.1 or 1 mM) referred to Extended Data Fig. 6g. **f**) qRT-PCR analysis of *Asns* expression in experimental replicates in MEF^{Ift88} and mIMCD3^{Ift88} relative to MEF^{Ctrl} and mIMCD3^{Ctrl} referred to Extended Data Fig. 6i. Data in dot and bar plots are mean ± SD. Statistical analysis: Student's unpaired two-tailed *t*-test or one-way ANOVA, followed by Tukey's multiple comparisons test; ns: not significant, *p < 0.05, **p < 0.01, ***p < 0.001 ,****p < 0.0001.

UNCROPPED SCANS OF BLOTS FOR SUPPLEMENTARY DATA OF:

Primary cilia sense glutamine availability and respond via asparagine synthetase

Maria Elena Steidl[1,2,*], Elisa A. Nigro[1,*], Anne Kallehauge Nielsen[1,2], Roberto Pagliarini[1], Laura Cassina[1], Matteo Lampis[1,3], Christine Podrini[1], Marco Chiaravalli[1], Valeria Mannella[4], Gianfranco Distefano[1], Ming Yang[5,6], Mariam Aslanyan[7], Giovanna Musco[8], Ronald Roepman[7], Christian Frezza[5,6] and Alessandra Boletta[1]

[1] *Molecular Basis of Cystic Kidney Disorders Unit, Division of Genetics and Cell Biology, IRCCS, San Raffaele Scientific Institute, Milan, Italy*

[2] *Ph.D Program in Molecular and Cellular Biology, Vita-Salute San Raffaele University, Milan, Italy*

[3] *Current address: Department of Biosystems Science and Engineering, ETH Zurich, Basel, Switzerland*

[4] *Center for Omics Sciences, IRCCS, San Raffaele Scientific Institute, Milan, Italy*

[5] *MRC, Cancer Unit Cambridge, University of Cambridge, Hutchison/MRC Research Centre, Box 197, Cambridge Biomedical Campus, Cambridge, CB2 0XZ, United Kingdom*

[6] *Current address: CECAD Research Center, Cologne, Germany*

[7] *Department of Human Genetics and Radboud Institute for Molecular Life Sciences, Radboud University Medical Center, Nijmegen, The Netherlands*

[8] *Biomolecular Nuclear Magnetic Resonance Unit, Division of Genetics and Cell Biology, IRCCS, San Raffaele Scientific Institute, Milan, Italy*

[*] *Equal Contributions*

Running Title: Cilia, Nutrient Sensing and Glutamine

Address Correspondence to: Alessandra Boletta, Division of Genetics and Cell Biology, San Raffaele Scientific Institute, Via Olgettina, 58, 20132, Milano, Italy. E-mail: boletta.alessandra@hsr.it

Uncropped Western blots of Supplementary Figures

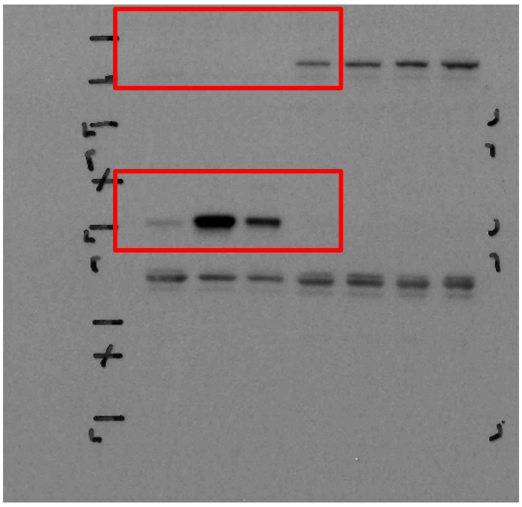

TSC1

Acetylated Tubulin

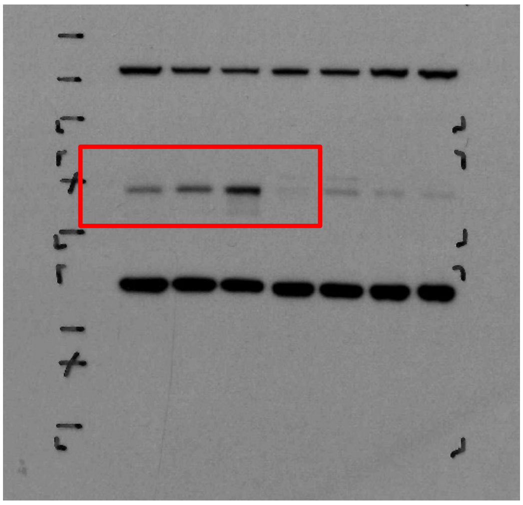

ASNS

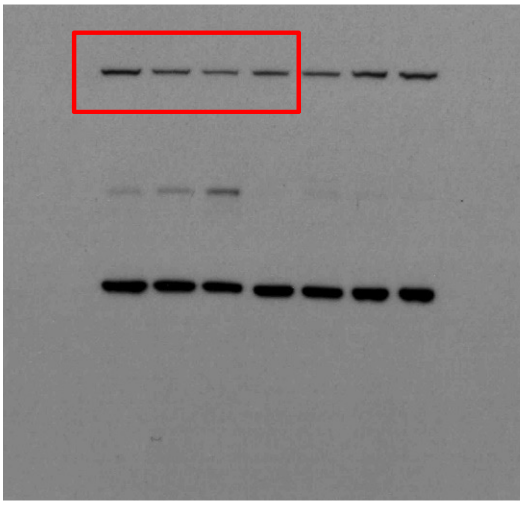

Vinculin

Supplemenatary Figure S7c
