# Supplementary material for: Primary cilia sense glutamine availability and respond via asparagine synthetase
Source: Nat Metab. 2023 Mar 6;5(3):385–97. doi: 10.1038/s42255-023-00754-6 (PMC10042734; doi:10.1038/s42255-023-00754-6)
Supplement: Supplementary file 2 — Reporting Summary [file 42255_2023_754_MOESM2_ESM.pdf]

nature portfolio

Corresponding author(s): Alessandra Boletta

Last updated by author(s): 20 January 2023

Reporting Summary

Nature Portfolio wishes to improve the reproducibility of the work that we publish. This form provides structure for consistency and transparency in reporting. For further information on Nature Portfolio policies, see our Editorial Policies and the Editorial Policy Checklist.

Statistics

For all statistical analyses, confirm that the following items are present in the figure legend, table legend, main text, or Methods section.

n/a	Confirmed	
☐	☒	The exact sample size (*n*) for each experimental group/condition, given as a discrete number and unit of measurement
☐	☒	A statement on whether measurements were taken from distinct samples or whether the same sample was measured repeatedly
☐	☒	The statistical test(s) used AND whether they are one- or two-sided<br>*Only common tests should be described solely by name; describe more complex techniques in the Methods section.*
☒	☐	A description of all covariates tested
☒	☐	A description of any assumptions or corrections, such as tests of normality and adjustment for multiple comparisons
☐	☒	A full description of the statistical parameters including central tendency (e.g. means) or other basic estimates (e.g. regression coefficient) AND variation (e.g. standard deviation) or associated estimates of uncertainty (e.g. confidence intervals)
☐	☒	For null hypothesis testing, the test statistic (e.g. *F*, *t*, *r*) with confidence intervals, effect sizes, degrees of freedom and *P* value noted<br>*Give P values as exact values whenever suitable.*
☒	☐	For Bayesian analysis, information on the choice of priors and Markov chain Monte Carlo settings
☒	☐	For hierarchical and complex designs, identification of the appropriate level for tests and full reporting of outcomes
☒	☐	Estimates of effect sizes (e.g. Cohen's *d*, Pearson's *r*), indicating how they were calculated

Our web collection on statistics for biologists contains articles on many of the points above.

Software and code

Policy information about availability of computer code

Data collection	No code required
Data analysis	• Heatmaps have been created by applying the heatmap function of Matlab® r2019a with colormap represented in logarithmic scale.<br>• Metabolic analysis have been obtained by a homemade Matlab® function for which the code is provided as source data (Matlab_Code_MetabolicData.m).<br>• MetaboAnalyst 5.0 has been employed for Metabolite Set Enrichment Analysis.<br>• For statistical analysis GraphPad Prism 8.2.1 and Matlab® were used using statistical analysis tool.

For manuscripts utilizing custom algorithms or software that are central to the research but not yet described in published literature, software must be made available to editors and reviewers. We strongly encourage code deposition in a community repository (e.g. GitHub). See the Nature Portfolio guidelines for submitting code & software for further information.

Data

Policy information about availability of data

All manuscripts must include a data availability statement. This statement should provide the following information, where applicable:

- Accession codes, unique identifiers, or web links for publicly available datasets
- A description of any restrictions on data availability
- For clinical datasets or third party data, please ensure that the statement adheres to our policy

All raw data related to the studies shown in Figures and Extended Data Figures are available as Source Data. The original TIFF files used for generating the raw data relative to quantification of ciliary length are available in Figshare at the link: https://doi.org/10.6084/m9.figshare.21922299.

Human research participants

Policy information about studies involving human research participants and Sex and Gender in Research.

Reporting on sex and gender	N/A
Population characteristics	N/A
Recruitment	N/A
Ethics oversight	N/A

Note that full information on the approval of the study protocol must also be provided in the manuscript.

Field-specific reporting

Please select the one below that is the best fit for your research. If you are not sure, read the appropriate sections before making your selection.

☒ Life sciences ☐ Behavioural & social sciences ☐ Ecological, evolutionary & environmental sciences

For a reference copy of the document with all sections, see nature.com/documents/nr-reporting-summary-flat.pdf

Life sciences study design

All studies must disclose on these points even when the disclosure is negative.

Sample size

Each study in vivo was samples onto cohorts of three mice and whenever this was sufficient to reach significance no additional animals were used in compliance with the 3R rule governing our Institutional work with animal models

For in vitro studies generally at least three wells per condition were utilized. For the cilia counting experiments at least three fields were acquired per each coverslip. All visible cilia were counted and measured in length in each field of each coverslip and combined to reach the n that is now indicated in brackets in each of the figures and shown as individual dots in the dotplots. For the seahorse analysis each cell line or condition was plated in multiple wells (indicated in each dot-plot experiment shown). As per manifacturer's indication, when a single well was an extreme outlier it was eliminated from the average (explaining the different n numbers in the different SeaHorse experiments, i.e. 5 vs 11 or 12 samples). Real time PCR analysis were performed in three biological replicates, each analyzed in two technical replicates and the average of technical replicates represented the individual biological replicate point.

Data exclusions

No data were excluded from the analysis. As indicated above, the measurements of OCR or ECAR in indivual wells using the SeaHorse instrument can be completely negative in which case the individual point is removed. The starting number of wells in our studies (i.e. 12) allows to have sufficient biological replicates for analysis even in these samples.

Replication

As a standard policy in our laboratory key findings are repeated in the hands of at least two individuals prior to submitting manuscripts. In this case, some key findings were repeated by four investigators independently (EAN, MES, AKN, ML). Only data for which all replication studies were successful are reported in this manuscript. Most of the shown data are the result of at least three independent experiments with the exception of the metabolic profiling and tracing studies for which one experiment with 5 biological replicates was performed (individual data shown). All replicates for all experiments shown in Figures and Extended data figures are reported in Supplementary Data Information.

Randomization

Mice were randomly utilized with respect to gender. No randomization was required because all treatments were performed on WT mice and the genetically modified mice were analyzed with intra-litter matching controls.

Blinding

In vivo experiments were not performed blindly because the same individual administering the fasting or glutamine administration was collecting and preparing the samples for analysis. The acquisition of the images for cilia quantification was performed blindly and randomly across the entire kidney tissues by a different individual. All analysis of ciliary length in vivo was carried out blindly by two individuals (E.A.N and L.C), both unaware of the treatment (carried out by M.C.).

Reporting for specific materials, systems and methods

We require information from authors about some types of materials, experimental systems and methods used in many studies. Here, indicate whether each material, system or method listed is relevant to your study. If you are not sure if a list item applies to your research, read the appropriate section before selecting a response.

Materials & experimental systems

n/a	Involved in the study
☐	☒ Antibodies
☐	☒ Eukaryotic cell lines
☒	☐ Palaeontology and archaeology
☐	☒ Animals and other organisms
☒	☐ Clinical data
☒	☐ Dual use research of concern

Methods

n/a	Involved in the study
☒	☐ ChIP-seq
☒	☐ Flow cytometry
☒	☐ MRI-based neuroimaging

Antibodies

Antibodies used

• rabbit ARL13B, Proteintech, #17711-1-AP
• mouse Acetylated alpha-Tubulin, Sigma-Aldrich, #T6793, Lot 059M4876V
• rabbit Pericentrin, Covance, #PRB-432C, Lot LN#14921602
• mouse γ-tubulin, Sigma-Aldrich, #T6557, clone GTU-88
• rabbit p-AMPK (Thr172), Cell Signalling Technology, #2535S, Lot 21
• rabbit AMPK, Cell Signalling Technology, #2532, Lot 19
• rabbit ASNS, abcam, #ab111873
• rabbit p-S6RP (s235/236, Cell Signalling Technology, #2211s, Lot 23
• rabbit S6RP, Cell Signalling Technology, #2217, Lot 7
• rabbit Hamartin/TSC1, Cell Signalling Technology, #4906
• rabbit IFT88, Proteintech, #13967-1-AP
• mouse Vinculin V284 antibody, Millipore, #05-386, clone V284
• DBA Rhodamine, Vector Laboratories, # RL-1032-2
• HRP conjugated secondary antibodies were from GE Healthcare: anti-rabbit IgG HRP linked, #934V, Lot 17402176; anti-mouse IgG HRP linked, #NA9310V, Lot 17453977; anti-rat IgG HRP linked (#NA935V), Lot 17344491
• Fluorochrome-conjugated secondary antibodies were from Thermo Fisher Scientific: goat anti-rabbit AlexaFluor 488, #A-21441; goat anti-mouse AlexaFluor 546, #A-11003; chicken anti-mouse AlexaFluor 594, #A-21201; goat anti-rabbit Alexa Fluor 647, #A-21244

Validation

All the antibodies were validated including proper negative controls. For Ift88, we used murine KO cells. For ASNS, murine knock-down cells. For TSC1, we used Tsc1 murine KO cells. For pAMPK and mTOR pathway treatment (pS6Rp) with appropriate activators or inhibitors was included in WBs in the same species that was used for the studies (mice). For Arl13b, cilia-less cells served as a negative murine control. For vinculin, acetylated tubulin, pericentrin mouse gamma tubulin, DBA rodamine no validation was performed as these are widely used by the scientific community. For HRP and fluorochrome secondary antibodies, staining in the absence of a primary antibody was used as a negative control.

Eukaryotic cell lines

Policy information about cell lines and Sex and Gender in Research

Cell line source(s)

• Mouse Embryonic Fibroblasts (MEFs) used are described in Distefano, G. et al. Mol Cell Biol 29, 2359-2371, doi:10.1128/MCB.01259-08 (2009).
• Madin-Darby Canine Kidney type II (MDCKII) cells are described in Boletta, A. et al. Mol Cell 6, 1267-1273, doi:10.1016/s1097-1028 2765(00)00123-4 (2000) and were originally acquired from the American Type Culture Collection (ATCC) by Dr. Lucia Monaco, Milan, Italy in 1996.
• Murine Inner Medullary Collecting Duct (mIMCD3) cells were kindly provided by Dr. Miriam Schmidts, Center for Pediatrics and Adolescent Medicine, Medical Center, University of Freiburg Freiburg, Germany.
• Human Retinal Pigment Epithelial (hRPE) cells were kindly provided by Dr. Nicoletta Landsberger, San Raffaele Scientific Institute, Milan, Italy.

Authentication

No authentication was performed

Mycoplasma contamination

All cell lines were tested negative for mycoplasma contamination

Commonly misidentified lines
(See ICLAC register)

None

Animals and other research organisms

Policy information about studies involving animals; ARRIVE guidelines recommended for reporting animal research, and Sex and Gender in Research

Laboratory animals	Wild type C57BL/6N mice; Opaflox/flox mice (kindly provided by Dr. Luca Scorrano, VIMM, Padua, Italy) and KspCre mice (Cadherin-16-Cre, kindly provided by Dr. Peter Igarashi, University of Minnesota, Minneapolis, USA) were inter-crossed to generate Opaflox/flox:KspCre experimental mice in a pure C57BL/6N genetic background (intra-litter Opaflox/+:KspCre or Opaflox/flox were used as controls)
Wild animals	The study did not involve wild animals
Reporting on sex	Study findings were not applied to only one sex: the female to male ratio was 1:1, Except for the Pharmacokinetics studies in which all the mice used were female. This because the dosage of glutamine was normalized for the weight and we wanted to decrease the variability of the groups for the final volume injected. Mice were randomized for each experiment. Research into sex was determined by the method of testis development recognition.<br>Total mice used: 46, divided into:<br>- Opa1 flox/flox at P2: 1 mutant vs 1 ct mouse;<br>- Opa1 flox/flox at P30: 3 mutant vs 3 cts mice;<br>- For the fasting studies: 24 wt mice, adults<br>- for the Pharmacokinetics studies: 14 wt mice (female), adults
Field-collected samples	The study did not involve samples collected from the field
Ethics oversight	All animal care and all protocols used were carried out according to the Institutional regulations and specifically approved by the institutional care and use ethical committee at the San Raffaele Scientific Institute, further approved by the Italian Ministry of Health (IACUC #921)

Note that full information on the approval of the study protocol must also be provided in the manuscript.
