# Supplementary material for: Primary cilia sense glutamine availability and respond via asparagine synthetase
Source: Nat Metab. 2023 Mar 6;5(3):385–97. doi: 10.1038/s42255-023-00754-6 (PMC10042734; doi:10.1038/s42255-023-00754-6)
Supplement: Supplementary file 14 — Uncropped western blots for Fig. 4b,h and Extended Data Figs. 1a,d, 3c and 6a,c. [file 42255_2023_754_MOESM14_ESM.pdf]

Uncropped Western blots

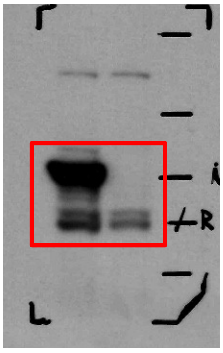

IFT88 MEF

Extended Data Figure 1a

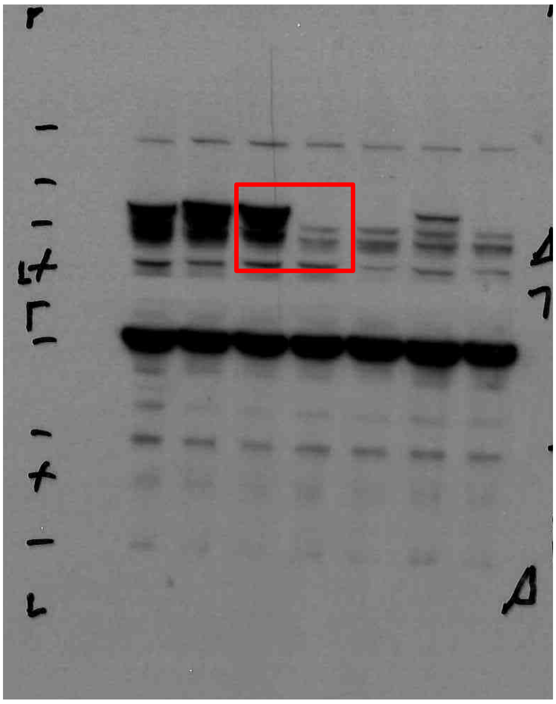

IFT88 IMCD

Extended Data Figure 1d

Uncropped Western blots

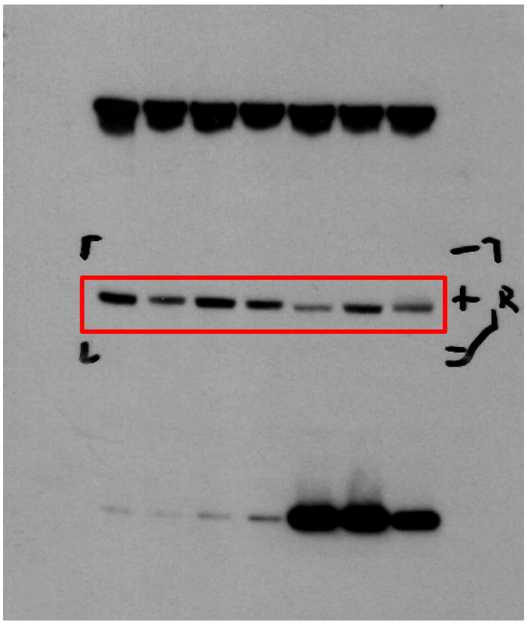

pAMPK

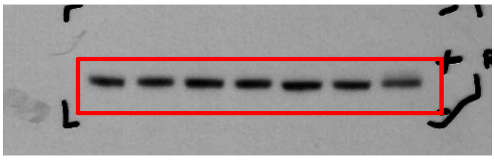

AMPK

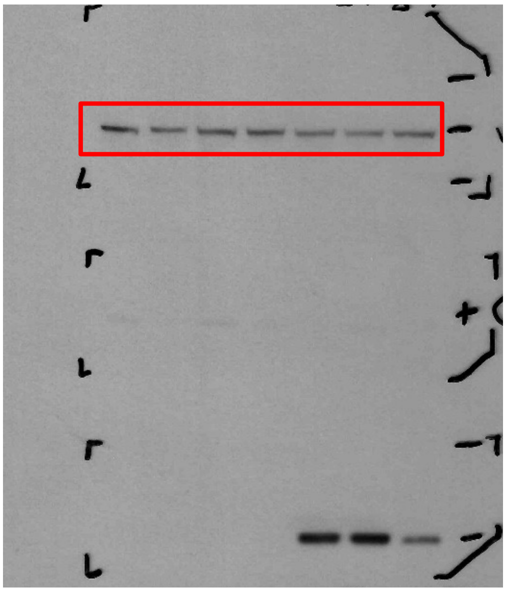

Vinculin

Extended Data Figure 3c

Uncropped Western blots

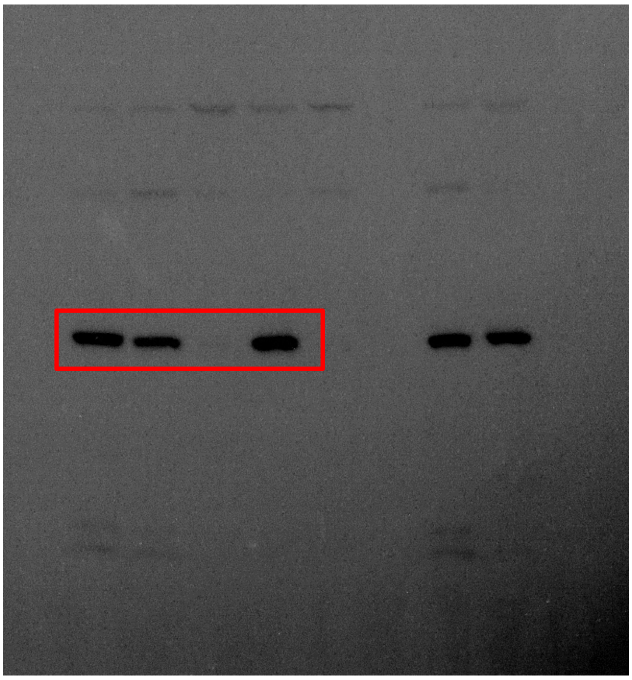

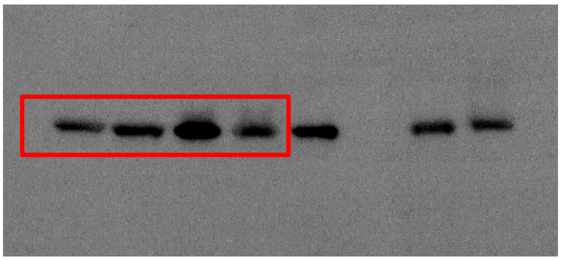

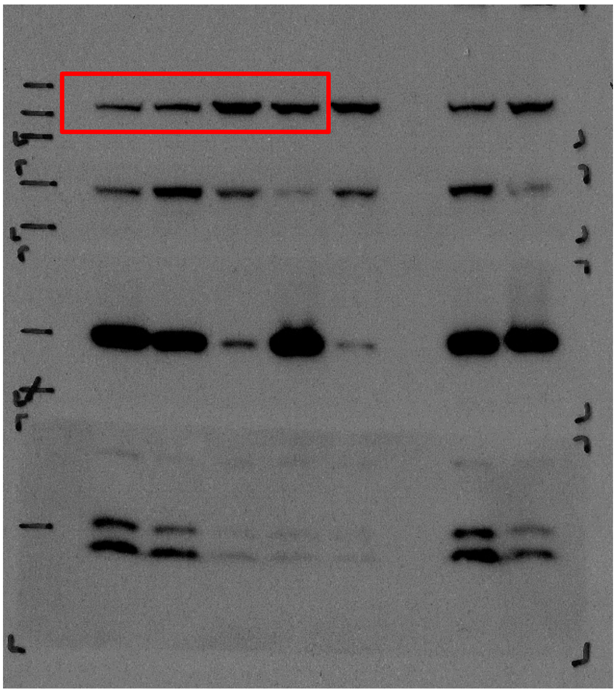

Extended Data Figure 6a

Uncropped Western blots

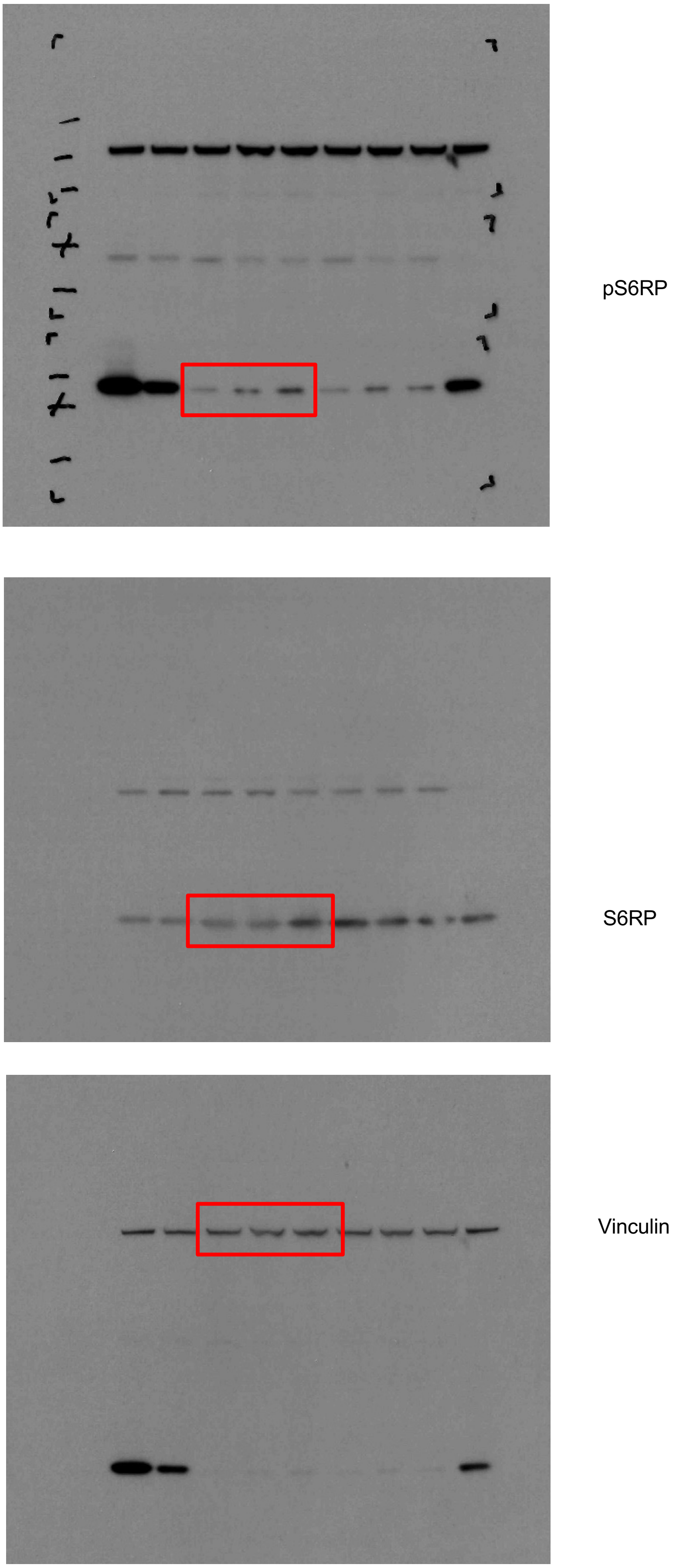

Extended Data Figure 6c

Uncropped Western blots

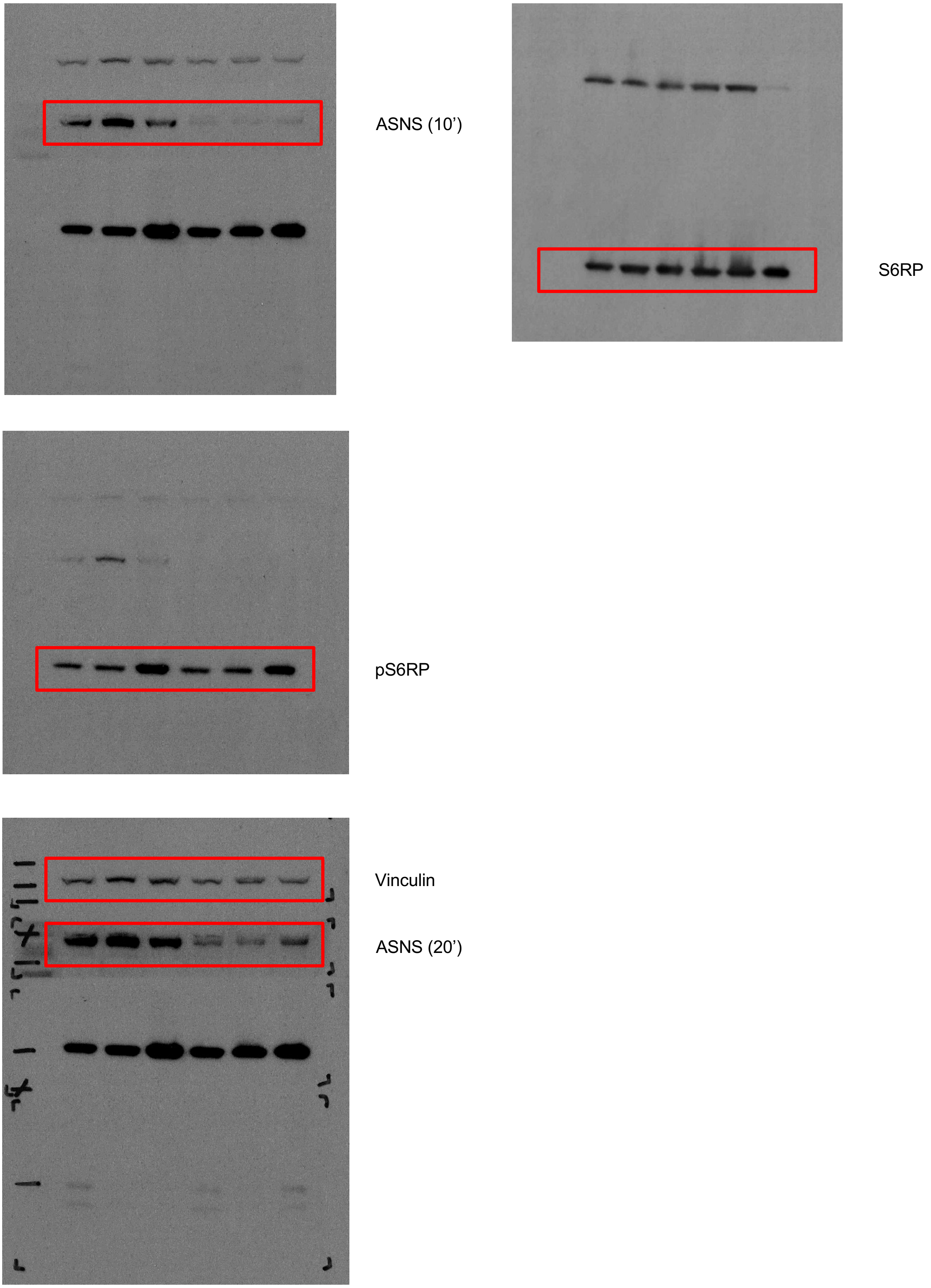

Figure 4b

Uncropped Western blots

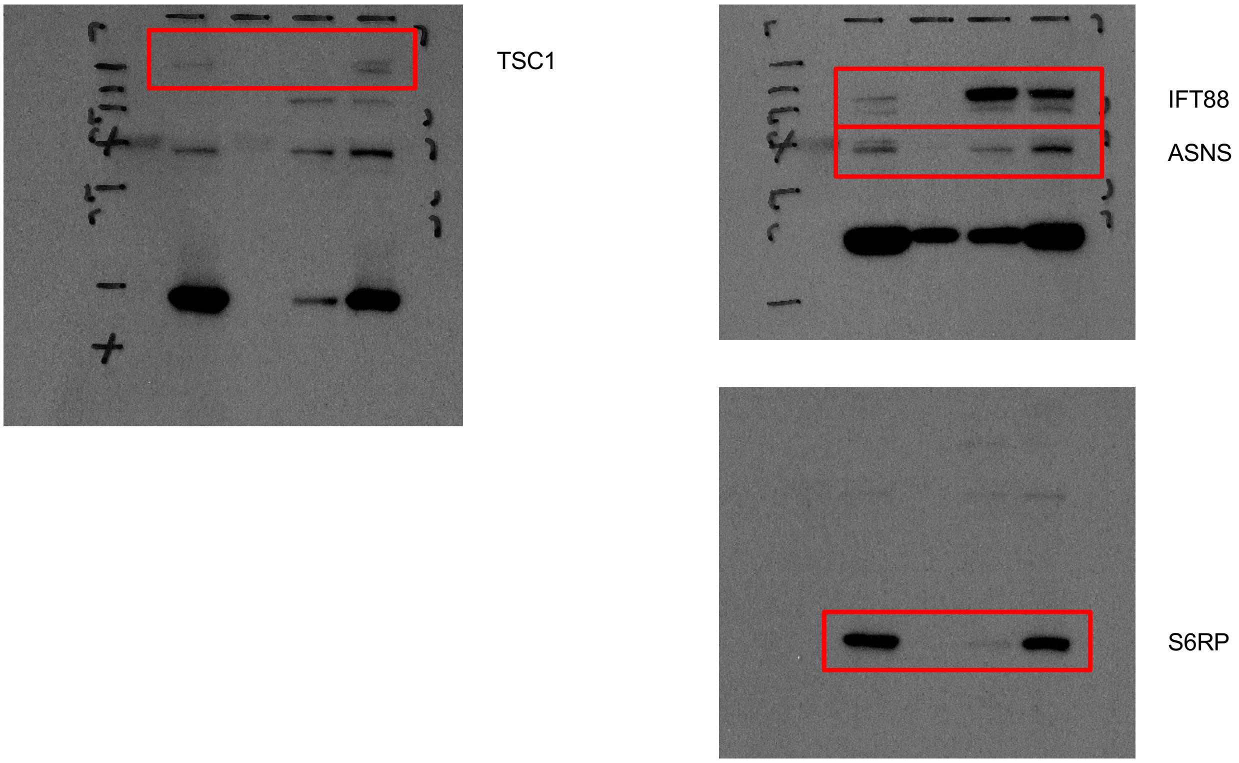

Figure 4h
